# Homocysteine and thyroid diseases

**DOI:** 10.3389/fendo.2025.1572997

**Published:** 2025-07-10

**Authors:** Lili Cui, Fei Wang, Chunyu Li, Fan Liu, Haipeng Wang, Junyu Zhao

**Affiliations:** ^1^ Department of Endocrinology and Metabology, The First Affiliated Hospital of Shandong First Medical University & Shandong Provincial Qianfoshan Hospital, Shandong First Medical University, Shandong Key Laboratory of Rheumatic Diseaseand Translational Medicine, Shandong Institute of Nephrology, Jinan, China; ^2^ College of First Clinical Medicine, Shandong University of Traditional Chinese Medicine, Jinan, China

**Keywords:** homocysteine, hyperhomocysteinemia, hypothyroidism, hyperthyroidism, MTHFR

## Abstract

Homocysteine (Hcy) is an important intermediate product in methionine metabolism which plays a key role in the methylation of DNA, RNA and proteins. High level of Hcy can induce endothelial cell damage, promote the release of inflammatory factors, stimulate oxidative stress and inhibit the fibrinolytic system. Numerous studies have confirmed the close relationship between hyperhomocysteinemia (HHcy) and the occurrence/development of various diseases such as cardiovascular diseases, neurological disorders, thrombotic diseases, and tumors. With the rising incidence of thyroid diseases, the relationship between Hcy and thyroid diseases has attracted widespread attention. It has been found that HHcy may be directly or indirectly associated with the development of hypothyroidism, but the findings with hyperthyroidism, chronic lymphocytic thyroiditis and reduced thyroid hormone sensitivity are controversial. This article reviews the research progress of Hcy and thyroid diseases, with a view to providing new ideas for the prevention and clinical treatment of diseases.

## 1 Introduction

In the 1930s, Nobel Prize-winning chemist *Vincent du Vigneaud* isolated homocysteine (Hcy) from bladder stones. Hcy is an intermediate metabolite of the methionine cycle which is a naturally occurring sulfur-containing amino acid with important roles in nucleic acid synthesis, DNA methylation, amino acid homeostasis, epigenetic maintenance, and redox processes. Normally, about 80% of Hcy is bound to plasma proteins such as albumin by disulfide bonds, and the rest of Hcy is mostly bound to thiols in the form of cystine or other forms, while only a small portion is free. Higher than normal level of Hcy in the blood is called hyperhomocysteinemia (HHcy). *The Expert Consensus on the Diagnosis and Treatment of Hyperhomocysteinemia* sets the diagnostic standard for HHcy at 10 umol/L. The consensus also points out that the median Hcy for adults in China is in the range of 13–14 umol/L, exceeding the diagnostic standard by more than 30% (1). With the gradual deepening understanding of Hcy, researchers have found that HHcy is closely related to the occurrence/development of cardiovascular diseases, stroke, diabetes mellitus complications, chronic kidney disease, pregnancy disorders, tumors and other diseases (1–3). Although the relationship between Hcy and thyroid diseases is not complete, changes in Hcy level are still considered by many scholars to be the cause or consequence of thyroid diseases. This article reviews the relationship between Hcy and thyroid diseases with the aim of providing new ideas for disease prevention, diagnosis and control.

## 2 Source and metabolism of Hcy

**Figure 1:**
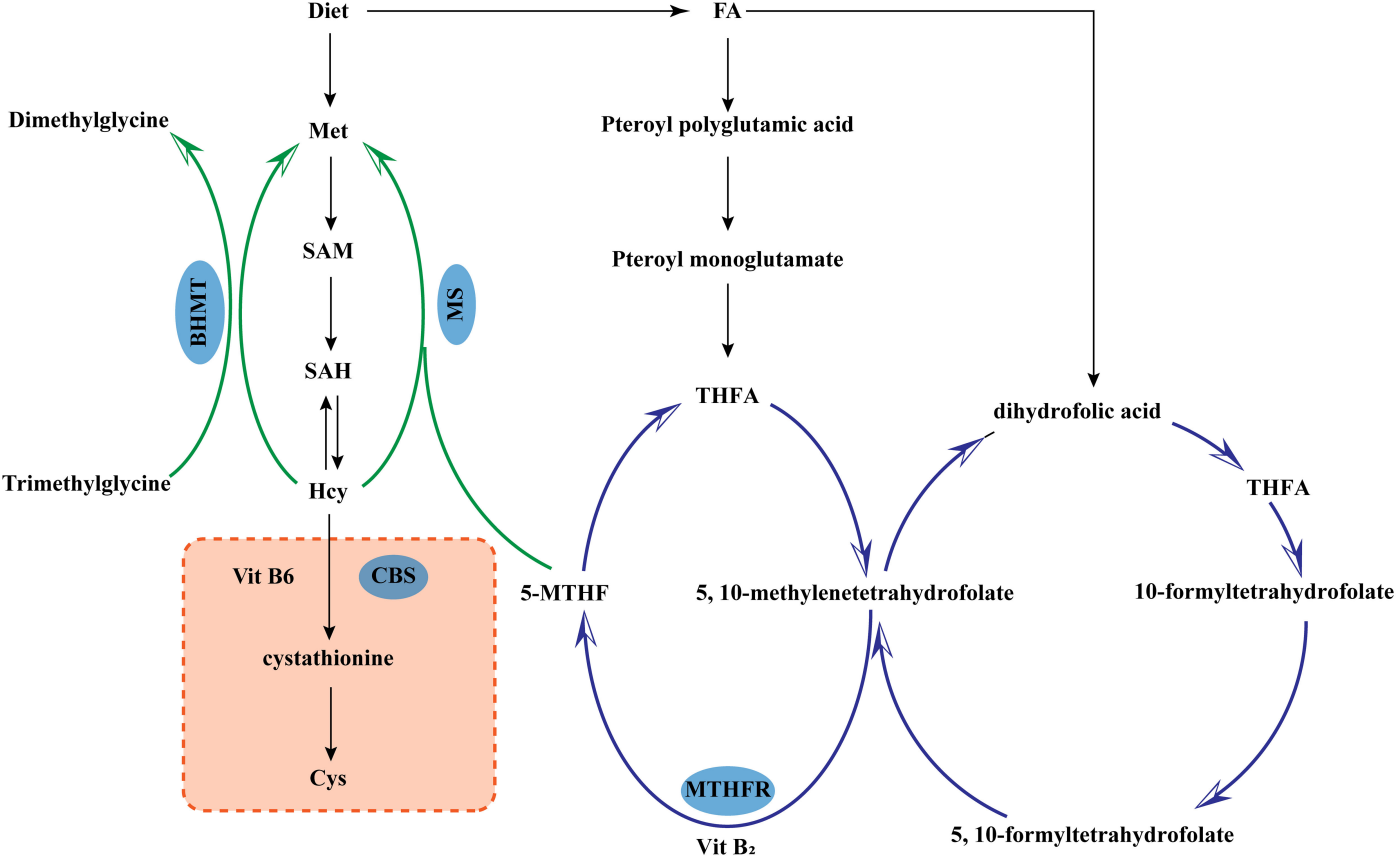
Metabolism of homocysteine. 5-MTHF, 5-Methyltetrahydrofolic acid; BHMT, Betaine-homocysteine methyltransferase; CBS, Cystathionine-β-synthase; Cys, L-Cysteine; FA, Folic acid; Hcy, Homocysteine; Met, Methionine; MS, Methionine synthase; SAM, S-adenosylmethionine; SAH, S-Adenosyl-L-homocysteine; MTHFR, Methylenetetrahydrofolate Reductase; THFA, Tetrahydro folic Acid.

Hcy is a product of demethylation during the methionine cycle, which is the only source of Hcy. Hcy is metabolized mainly through the remethylation pathway and the transsulfuration pathway (4). The two pathways coexist and complement but cannot completely replace each other. Only when the two metabolic pathways are in balance can Hcy be kept within the normal range (**Figure 1**). (1) Remethylation can occur in cells of a variety of tissues. Hcy meets with the methyl group provided by 5-Methyltetrahydrofolic acid (derived from folic acid) or betaine to produce methionine catalyzed by methionine synthase (MS). (2) Transsulfuration which is the production of cysteine by Hcy with the assistance of vitamin B6, catalyzed by cystathionine-β-synthase (CBS). Cysteine can form disulfide bonds which have the effect of stabilizing the spatial structure of peptide chains.

## 3 Influencing factors of Hcy level

### 3.1 Genetic factors

Genetic errors or deficiencies in key enzymes of Hcy metabolism can lead to HHcy. Methylenetetrahydrofolate reductase (MTHFR) C677T mutation is the most common cause. The mutations can lead to a decrease in the activity and heat resistance of the enzyme, which in turn affects the cycling of folic acid (FA). It was found that in CT and TT populations, the activity of MTHFR was only 65% and 30% of that in CC populations. On the other hand, Hcy levels were increased by 20%-70% in TT populations compared to CC populations (5, 6) (**Supplementary Table 1**).

### 3.2 Age and gender

Conventional opinion is that Hcy level increases with age (7). But in recent years, studies have found that there is a trend towards a younger age for HHcy, which has become an independent risk factor for cardiovascular and cerebrovascular diseases in young and middle-aged people (8). Hcy levels are significantly higher in men than in women due to the important role of sex hormones in methionine metabolism (9).

### 3.3 Dietary and nutritional factors

Long-term consumption of protein leads to excessive intake of methionine which in turn raises Hcy level, while eating large amounts of vegetables and fruits can indirectly reduce Hcy level by increasing vitamin content. Bad lifestyles such as prolonged heavy drinking, coffee consumption or smoking can lead to elevated Hcy level by reducing intake or increasing consumption of B vitamins and FA.

(4) Diseases: Chronic kidney disease, severe anemia, hypothyroidism, malignancy and drugs that affect the absorption of B vitamins and FA have been shown to increase Hcy level (10).

## 4 Hcy and thyroid hormones

Thyroid hormone (TH) is a major regulator of cellular metabolism and maintains metabolic homeostasis by coordinating networks of carbohydrate, lipid and protein metabolism. In tissues, thyroid hormone action is mediated by transmembrane transporters, deiodinases and thyroid hormone receptors. The selenoprotein family of iodothyronine deiodinases consists of three enzymes, D1, D2 and D3, which are specifically distributed in tissues and regulate the activation and inactivation of TH: type 1 and 2 deiodinases (D1/D2) catalyse the conversion of the inactive precursor thyroxine (T4) to the active form, triiodothyronine (T3), while type 3 deiodinases (D3) terminate the hormone’s action by degradation (11, 12). Studies have shown that homocysteine (Hcy) can impair deiodinase activity through oxidative stress and inflammatory mechanisms. In addition, studies have highlighted a biochemical link between Hcy metabolism and glutamine metabolism that may be mediated by stress-related pathways affecting cellular function (13). The critical role of thyroid hormones (THs) in regulating glutamine metabolism has been demonstrated (14). Together, these findings suggest that THs may constitute an important metabolic regulatory network involved in stress response and cellular function through direct regulation of glutamine metabolism, which may in turn indirectly influence Hcy metabolic pathways.

## 5 Hcy and thyroid diseases

### 5.1 Hcy and thyroid nodules

In recent decades, with the widespread availability of ultrasound technology, thyroid nodules have become a common clinical disease. The prevalence of thyroid nodules with a diameter of more than 0.5 cm in Chinese adults is as high as 20.43% (15). Current studies have found that the development of thyroid nodules is associated with genetic factors, gender, age, iodine intake, autoimmunity and insulin resistance. However, Hcy and thyroid nodules have been less studied (16–19). By analyzing the serological indicators of 48 patients with thyroid nodules and 52 healthy people, Li et al. found that the Hcy levels of patients with thyroid nodules were significantly higher (*P < 0.05*) (16). Clinical studies have found a positive correlation between Hcy level and the prevalence of thyroid nodules, which is an independent risk factor for the development of thyroid nodules (19, 20). In patients with type 2 diabetes mellitus (T2DM), high level of Hcy increases the incidence of thyroid nodules by 1.055-1.475 times (17). The reason for this phenomenon may be related to the fact that Hcy affects cell methylation and regulates cell proliferation and apoptosis (21). On the other hand, Hcy is associated with autoimmune response and insulin resistance, which are also important etiological factors that induce thyroid nodules (22, 23). In conclusion, Hcy may be an important indicator for thyroid nodule surveillance. Thyroid ultrasound screening of patients with high level of Hcy may facilitate early detection of thyroid nodules.

### 5.2 Hcy and hyperthyroidism

Hyperthyroidism is a group of clinical syndromes in which increased thyroid hormones by the thyroid gland cause accelerated metabolism in the body. In a hypermetabolic state, the body releases large amount of reactive oxygen species (ROS), which can damage thyroid follicular cells, release intracellular antigens to activate the immune system and induce autoimmune deterioration. Nedrebø et al. reported no significant difference in Hcy levels between hyperthyroid patients and controls, whereas Demirbaş et al. found reduced serum Hcy levels in untreated hyperthyroid patients (24, 25). This difference may be attributed to increased renal clearance of Hcy due to increased glomerular filtration rate in hyperthyroid states. Another important factor is the regulation of several enzyme systems by thyroid hormones, which may affect the activity of enzymes involved in the Hcy metabolic pathway. Moreover, A study by Nechiporuk found that hyperthyroidism elevated the activities of CBS, cysteine dioxygenase (CDO), sulfite oxidase (SO) in brain and cysteine aminotransferase (CAT) in heart, which accelerated the process of transsulfuration (26). This seems to explain why researchers often discover the phenomenon that Hcy levels are reduced in patients with hyperthyroidism (27–31). Interestingly, findings regarding changes in Hcy levels before and after treatment for hyperthyroidism patients have not always been consistent. Studies by Pawilojc and Nedrebø both found that Hcy level gradually rebounded as the disease resolved (32, 33). On the contrary, in Colleran’s study, Hcy levels were further reduced in Graves’ disease patients after treatment with methimazole (34). Why it occurs is unclear and may be related to the immunomodulatory properties of methimazole as well as organismal immune recovery (35, 36). The results of current studies point to a reduction of Hcy level in patients with hyperthyroidism, but relationships between the condition and the change in Hcy level remain divergent and require more researchs.

### 5.3 Hcy and hypothyroidism

#### 5.3.1 Hcy and clinical hypothyroidism

Elevated Hcy levels in patients with clinical hypothyroidism have been generally recognized by researchers at home and abroad (37–40), and some researchers have suggested that Hcy can be used as a diagnostic reference indicator for hypothyroidism. Impaired Hcy conversion due to decreased activity of metabolically key enzymes such as MTHFR and reduced absorption of nutrients from the gastrointestinal tract are the main reasons for elevated Hcy level (41, 42). On the other hand, lowered glomerular filtration rate or even possible immune complex nephritis that reduces Hcy clearance could also explain the elevated Hcy (43). Hcy is an important risk factor for cardiovascular diseases. The risk of developing coronary heart disease was found to increase by 40% for every 4 umol/L increase in Hcy (44). A study by Dang et al. found that elevated Hcy was associated with the development of myocardial injury and left ventricular hypoplasia in patients with hypothyroidism (37), which may be related to the activation of mitochondrial matrix metalloproteinases by Hcy. Hcy increases oxidized low-density lipoprotein (OxLDL) and causes endothelial cell dysfunction, which in turn accelerates arterial inflammation and fat deposition (39, 40, 45). From another perspective of lipid metabolism, Hcy inhibits the expression and function of apolipoprotein A-I to the extent that circulating high-density lipoprotein (HDL) level is reduced (46–49). Altogether, Hcy can participate in the atherosclerotic process through various mechanisms such as inducing mitochondrial dysfunction and promoting lipid peroxidation. This implies that patients with hypothyroidism who have a combination of elevated Hcy are at a substantially higher risk of cardiovascular diseases, and that attention to Hcy level can be useful in assessing disease risk. Hormone replacement therapy is a routine treatment strategy for patients with hypothyroidism. Several studies have confirmed that levothyroxine (L-T4) can reduce Hcy level (50). In addition, Catargi et al. reported lower levels of folic acid in hypothyroid patients and recommended supplementation of folic acid along with thyroid therapy (51). Amir Ziaee also noted that the combination of LT4 and folic acid is more effective in lowering serum homocysteine levels (52). Therefore, clinical hypothyroid patients should be actively tested with Hcy and treated in a timely manner.

#### 5.3.2 Hcy and subclinical hypothyroidism

Only thyroid stimulating hormone (TSH) level is elevated, while free triiodothyronine (FT3) and free tetraiodothyronine (FT4) level remain within the normal range are important features of subclinical hypothyroidism (SCH). Epidemiological data showed that the global prevalence of SCH was approximately 1.3%-10%. The incidence of SCH increases progressively with age. The prevalence of SCH in the elder group can be as high as 5.7%-20% (53). Studies on the natural history of the disease suggest that SCH is more likely to progress to clinical hypothyroidism when combined with thyroid autoantibody positivity or TSH >10 mU/L, and thus SCH is also considered to be a precursor to clinical hypothyroidism.

Based on the correlation between Hcy and clinical hypothyroidism, more researchers have shifted their attention to the relationship between Hcy and SCH with a series of clinical studies (54–57), but the results of these studies are not the same. Aldasouqi et al. tested Hcy levels in 47 patients with SCH and did not find correlations (55). In another study, researchers divided SCH patients into two groups based on TSH levels and found that only SCH patients with TSH >10 mU/L had high Hcy levels (57). Data from Wang et al. suggested that regardless of the extent of SCH disease, Hcy levels were elevated in patients compared to the normal group (*P < 0.05*), and Hcy levels were higher in patients with severe disease compared to those with mild (*P < 0.05*) (54). A Meta-analysis of 12 observational studies conducted by Zhang showed that SCH patients had high Hcy levels compared to subjects with normal thyroid function (58). The study also pointed out that differences in testing techniques could affect research on the correlation between Hcy and subclinical hypothyroidism, which may also account for the different results of numerous studies. On the other hand, Hcy is also an independent risk factor for SCH which is involved in the occurrence and development of SCH (59). Thyroid peroxidase (TPO) is an autoantigen that catalyzes thyroid hormones (60, 61). Hcy increases the expression of thyroid peroxidase antibody (TPOAb), which inhibits thyroid function by decreasing TPO activity and exacerbates SCH (62).

In addition to exacerbating cardiovascular burden, Hcy is directly related to insulin resistance (IR) by inducing secretion and expression of resistin (63, 64). The prolonged high Hcy state of the body aggravates the risk of diabetes mellitus in SCH patients (65, 66). Considering that Hcy level is closely related to the severity and natural prognosis of SCH, some scholars believe that Hcy is a predictor of SCH patients’ prognosis. An observational study that included 104 patients with SCH found that Hcy level can predict the prognosis of SCH patients with area under curve (AUC) as high as 0.946 by ROC curve plotting (54). Hcy levels were positively correlated with SCH condition and SCH patients with HHcy had a higher risk of progressing to clinical hypothyroidism. Similar to the effect of the intervention in patients with clinical hypothyroidism, Hcy levels in patients with SCH can be significantly reduced after L-T4 replacement therapy (40).

Although the change of Hcy level in SCH patients remains controversial, high level of Hcy is often seen in SCH patients. Increased Hcy level can be both a consequence of SCH and a risk factor for SCH. The changes of Hcy level is associated with the development of other diseases such as cardiovascular disease and insulin resistance in patients with SCH, which can be used as a prognostic indicator for SCH. Hcy should be monitored dynamically in SCH patients.

#### 5.3.3 Hcy and hypothyroidism in pregnancy

Data from epidemiological surveys showed the prevalence of clinical hypothyroidism in pregnancy was 0.3%-1.9% and the prevalence of subclinical hypothyroidism in pregnancy was 1.5%-5% (67, 68). While analysis of epidemiological data in China revealed that the prevalence of clinical hypothyroidism in pregnancy and subclinical hypothyroidism in pregnancy were respectively 1% and 5.27% (69, 70). Increased iodine demand, increased renal iodine clearance, compensatory enlargement of the thyroid gland and change in sex hormone are important causes of hypothyroidism in pregnancy (71, 72). Moreover, other studies have found that the activated immune system of the maternal body during pregnancy induces the production of TPOAb, which damages the thyroid gland and induces hypothyroidism (73). Hypothyroidism in pregnancy is not only strongly associated with many complications such as gestational hypertension and gestational diabetes mellitus, but also raises the risk of adverse pregnancy outcomes such as miscarriage, preterm labor, intrauterine growth and developmental abnormalities in the fetus (74–76).

Hcy levels in healthy pregnant women begin to decline in early stage and can fall to 50%-60% of pre-pregnancy levels by 20–32 weeks, remaining relatively stable or rising slightly in late pregnancy (77). This may be related to changes in hormones, increased blood volume, increased glomerular filtration rate, increased amino acid requirements of the pregnant woman and fetus during pregnancy. Low level of Hcy maintains the integrity and function of maternal vascular endothelial cells, which helps to regulate the adaptation to pregnancy. On the other hand, it also ensures the normal development of multiple organs or systems, including nervous system.

Several studies have found that Hcy levels are significantly elevated in women with hypothyroidism in pregnancy which is positively correlated with TSH levels (78–82). In addition to the reasons already analyzed before, the increased demand for vitamins and FA by pregnant women and changes in hormones are also key reasons for the rise in Hcy. A study by Zhang et al. found a significant negative correlation between folate level and Hcy level (83), which supported the idea that a relative deficiency of FA was responsible for elevated Hcy level. HHcy is strongly linked to adverse pregnancy outcomes through its engagement in vasculopathy processes such as placental vascular micro thrombosis and induction of abnormal fetal growth and development (80, 81, 84, 85). Meanwhile, HHcy promotes the release of inflammatory factors, stimulates oxidative stress and increases the maternal cardiovascular burden. Obvious improvements in thyroid function and Hcy were seen in hypothyroidism patients with pregnancy who were treated with L-T4 (86). However, its effect on pregnancy outcomes is uncertain. A study of infertile women with SCH showed that L-T4 treatment before and after assisted reproductive technology could reduce the rate of miscarriage and increase the rates of embryo implantation (87). A Meta-analysis by Rao et al. in 2018 found that L-T4 treatment only reduced the miscarriage rate, with no significant effect on the clinical pregnancy rate, live birth rate or preterm birth rate (88). This is probably related to the fact that only four randomized controlled trials were included in the study by Rao. The timing of thyroid function tests and LT4 treatment varied from the different studies.

Although there are conflicting views on the benefits of L-T4 supplementation in hypothyroidism patients with pregnancy, it is generally considered to be useful in reducing the rate of miscarriage. Women with hypothyroidism during pregnancy should be monitored for changes in Hcy, folic acid and vitamins.

### 5.4 Hcy and chronic lymphocytic thyroiditis

The incidence of CLT has been increasing with the widespread of screening tests. However, epidemiological data on CLT are still limited due to its atypical clinical symptoms in that a large number of patients who have not yet developed to thyroid function abnormalities are not diagnosed. There is a bidirectional link between Hcy and immune inflammation. In the first place, Hcy is an initiator of autoimmune and inflammatory, which induces the transcription of multiple cytokines and proinflammatory mediators. Alternatively, elevated Hcy is a consequence of immune and inflammation, which stimulates oxidative stress and increases antioxidant depletion.

The study by Cicone included patients with acute medical hypothyroidism and analyzed their Hcy levels. Hcy levels were found to be markedly higher in patients with Hashimoto’s thyroiditis than non-Hashimoto’s thyroiditis patients (89). Fewer studies have been reported on Hcy in CLT patients with thyroid dysfunction. Only a part of the studies has seen elevated Hcy levels in thyroglobulin antibody (TgAb)-positive or TPOAb-positive subjects (90). These findings suggest that alterations in Hcy levels of CLT patients may be related to autoimmunity. The concentration of TPOAb is directly tied to the degree of lymphocyte infiltration within the thyroid gland (91, 92). Even if the disease has not progressed to thyroid dysfunction, the presence of TPOAb can influence Hcy level through constantly stimulating the immune-inflammatory system. Previous studies have found that thyroid autoantibodies are associated with nutrients such as FA and vitamins (90, 93), so some researchers have also hypothesized that the relationship between thyroid autoantibodies and Hcy may be mediated by FA and vitamins. Similar to the findings in SCH, elevated Hcy can damage the thyroid gland by enhancing the activity of TPOAb. At the same time, higher Hcy level is directly related to the risk of several diseases, including cardiovascular diseases (94).

There are still no precise conclusions about the relationship between Hcy and CLT without thyroid dysfunction, which still needs to be confirmed by large sample and mechanistic studies.

### 5.5 Hcy and thyroid neoplasms

Thyroid cancer is the most common tumor of the endocrine system. The new incidence rate of thyroid cancer in China ranked the third highest among all cancer types in 2022 (95). The study of the pathogenesis of thyroid cancer has been the focus of many researchers. Hcy accumulated in the body synthesizes methionine through the remethylation pathway, which provides methyl for methylation of DNA, RNA and proteins. DNA methylation has been found to be an important contributor to the increased risk of thyroid cancer (96). In addition to affecting DNA methylation, high levels of Hcy upregulate histone H3K79Hcy, which regulates the expression levels of BMP7, CTNNB1, GLI2, NOTCH1, and RXRA genes (97). High expression of these genes has been reported to be associated with the development and progression of thyroid cancer (98–102). In addition, polymorphisms in the MTHFR gene, a key enzyme involved in folate metabolism, increase the risk of thyroid cancer (103). On the contrary, rapid proliferation of metabolic tumor cells increases the consumption of folic acid and vitamins, and the lack of folic acid and vitamins in the body will cause the elevation of Hcy, resulting in a vicious circle. Hcy can activate immunoinflammation, and immunoinflammation induces the development and progression of various tumors, so the specific mechanism of the role of Hcy in the development and progression of thyroid cancer needs to be further researched.

### 5.6 Hcy and reduced thyroid hormone sensitivity

Reduced responsiveness of tissue cells to thyroid hormones is seen in some normal populations in clinical work, with the coexistence of high FT4 combined with high TSH as the main manifestation, which may be related to impaired thyroid hormone sensitivity and can further develop into thyroid dysfunction (hyperthyroidism or hypothyroidism). A study by Ding et al. revealed for the first time that impaired thyroid hormone sensitivity can lead to elevated Hcy levels, and that reduced thyroid hormone sensitivity increases the risk of atherosclerosis, even during phases of normal thyroid function (104).

Thyroid hormones are involved in maintaining the activity of riboflavin metabolizing enzymes (especially riboflavin kinase). Flavin adenine dinucleotide is a cofactor of MTHFR, so thyroid hormones can influence the process of Hcy metabolism (105). When tissue thyroid hormone sensitivity is impaired, MTHFR activity is limited, which in turn increases Hcy levels. On the other hand, high levels of Hcy can regulate the expression of thyroid-related genes in the brain and thus affect the thyroid hormone network, further exacerbating thyroid hormone resistance (81).

The relationship between thyroid function and Hcy is not described in detail in this article because thyroid disorders are often combined with abnormal thyroid hormone sensitivity, the results of studies on the relationship between thyroid function and Hcy are conflicting, and we believe that the association between the two needs to be considered individually. The relationship between thyroid hormone sensitivity and Hcy needs to be confirmed by studies with large sample data.

## 6 Conclusion

There is a bidirectional link between thyroid disease and Hcy levels. Thyroid diseases tend to cause changes in Hcy levels, which in turn exacerbate the progression of the disease. High levels of Hcy have been found to be associated with the incidence of thyroid nodules, hypothyroidism, and thyroid cancer; hypothyroidism in turn can cause patients’ Hcy levels to rise further, increasing the risk of cardiovascular and cerebral vascular disease, forming a vicious cycle; while Hcy levels commonly fall in patients with hyperthyroidism. Clinically, thyroid function and ultrasound should be performed in a timely manner in people with changes in Hcy levels for early detection of disease and intervention. Further experimental and clinical studies are needed to confirm the role and biological mechanism of Hcy in the development of uncomplicated thyroid dysfunction CLT and thyroid cancer.
