# Supplementary material for: Homocysteine and thyroid diseases
Source: Front Endocrinol (Lausanne). 2025 Jul 10;16:1572997. doi: 10.3389/fendo.2025.1572997 (PMC12286808; doi:10.3389/fendo.2025.1572997)
Supplement: Supplementary file 1 [file Table1.docx]

**Supplemental Table 1. Genotype distribution frequency of MTHFR C677T polymorphism.**

|  | CC | CT | TT |
| --- | --- | --- | --- |
| Xiaojuan Zhao, 2024 [1] | 37.10% | 50.00% | 12.90% |
|  | 30.36% | 41.07% | 28.57% |
| G. RAVAGLIA, 2004 [2] | 29% | 50% | 21% |
| P. FERRONI, 2009 [3] | 36% | 41% | 23% |
|  | 38% | 53% | 9% |
| Jayantee Kalita, 2020 [4] | 91% | 9% | 0% |
|  | 52.5% | 30% | 17.5% |
| Can Wen, 2015 [5] | 25.9% | 30.4% | 43.7% |
|  | 40.7% | 45.9% | 13.4% |
| Silpita Paul, 2018 [6] | 59.7% | 29% | 11.3% |
|  | 78.1% | 21.9% | 0.0% |
| Jingyuan Zhang, 2022 [7] | 33.33% | 50.62% | 16.05% |
|  | 50.38% | 42.75% | 6.87% |
| Victoria I. Lioudyno, 2024 [8] | 62.75% | 31.37% | 5.88% |
|  | 49.02% | 45.10% | 5.88% |
| Irena Zuntar, 2006 [9] | 45% | 50% | 5% |
|  | 47% | 45% | 8% |
| KK Sukla, 2011 [10] | 76.72% | 21.18% | 2.10% |
| Nadia Koubaa, 2010 [11] | 61.9% | 30.5% | 7.6% |
|  | 50.5% | 33% | 16.5% |
| Luwen Huang, 2022 [12] | 60.12% | 36.90% | 2.98% |
|  | 47.47% | 36.36% | 16.16% |
| Varinderpal S.Dhillon, 2007 [13] | 35% | 50% | 15% |
|  | 45.25% | 43.02% | 11.73% |
| Dong Guan, 2023 [14] | 15.74% | 44.53% | 39.73% |
|  | 22.52% | 50.57% | 26.91% |
| Yoshiaki Somekawa, 2001 [15] | 44.24% | 41.94% | 13.82% |
| Huihui Guo, 2025 [16] | 27.9% | 48.4% | 23.8% |
|  | 16.4% | 31.8% | 51.8% |
|  | 34.8% | 58.4% | 6.9% |
| Fabio Coppede, 2012 [17] | 26.2% | 51.0% | 22.8% |
|  | 34.7% | 46.6% | 18.7% |
| Lin Jiajin, 2018 [18] | 39% | 51% | 10% |
|  | 55% | 37% | 8% |
| Sara Senemar, 2013 [19] | 51.1% | 38.1% | 10.8% |
|  | 58% | 34% | 8% |
| Bruno Zappacosta, 2013 [20] | 20.8% | 49.8% | 29.4% |
| Arthur Clément, 2019 [21] | 44.4% | 44.2% | 11.4% |
| Betti Giust, 2007 [22] | 24.7% | 52.5% | 22.8% |
|  | 29.2% | 48.7% | 22.1% |
| Y O¨ zkul, 2005 [23] | 29.9% | 59.1% | 11.0% |
|  | 35.7% | 61.9% | 2.4% |
| Hideki Kimura, 2000 [24] | 37.1% | 45.2% | 17.6% |
|  | 35.7% | 49.0% | 15.4% |
| Yan Long, 2019 [25] | 38.1% | 41.1% | 20.8% |
|  | 19.7% | 42.4% | 37.9% |
| Aifan Li, 2017 [26] | 23.7% | 44.7% | 31.6% |
|  | 40.6% | 42.2% | 17.2% |
| Xiaoxia Du, 2020 [27] | 1.7% | 16.9% | 81.4% |
|  | 8.7% | 43.7% | 47.6% |
| Nathalie M. J. van der Put, 1998 [28] | 44.09% | 41.94% | 13.98% |
| Vanessa Cavalcante da Silva, 2006 [29] | 44.2% | 44.2% | 11.6% |
|  | 50.0% | 46.0% | 4.0% |
| George V.Z. Dedoussis, 2005 [30] | 41.46% | 48.08% | 10.45% |
| Yongxin Liu, 2024 [31] | 40.44% | 49.63% | 9.93% |
|  | 35.06% | 49.89% | 15.06% |
| Weijuan Cai, 2014 [32] | 19.5% | 49.5% | 31.0% |
|  | 30.5% | 44.5% | 25.0% |
| Can Sun, 2023 [33] | 20.70% | 57.00% | 22.20% |
|  | 7.40% | 41.20% | 51.50% |
| Zhican Li, 2024 [34] | 2.1% | 14.6% | 83.3% |
|  | 8.0% | 45.1% | 46.9% |
| Shaoyan Zhang, 2019 [35] | 18.63% | 48.46% | 32.91% |
|  | 23.01% | 49.06% | 27.93% |
| Ningning Hou, 2016 [36] | 33.8% | 51.3% | 14.9% |
|  | 41.1% | 41.1% | 17.8% |
| K. T. Moe, 2008 [37] | 60.8% | 30.0% | 9.2% |
|  | 65.7% | 32.9% | 1.4% |
| Heming Wu, 2022 [38] | 50.87% | 42.10% | 7.03% |
|  | 57.07% | 36.84% | 6.09% |
| Ruilian Liang, 2014 [39] | 61.4% | 33.2% | 5.4% |
| Jose T Real, 2009 [40] | 34.40% | 44.00% | 21.60% |
| Minal Umesh Paradkar, 2019 [41] | 74.2% | 19.4% | 6.5% |
| Xin He, 2022 [42] | 50% | 40% | 10% |
| Luting Chen, 2021 [43] | 32.0% | 47.1% | 20.9% |
| Elias E. Mazokopakis, 2023 [44] | 19.06% | 52.74% | 28.20% |
| Sultan Mehmood Siddiqi, 2024 [45] | 28.0% | 45.9% | 26.1% |
| Zhao Li, 2015 [46] | 30.23% | 41.86% | 27.91% |
|  | 35.25% | 52.46% | 12.29% |
|  | 28.13% | 44.38% | 27.49% |
|  | 31.76% | 53.53% | 14.71% |
| Can Cai, 2016 [47] | 15.7% | 46.7% | 37.6% |
| Jamal Golbahar, 2004 [48] | 60.15% | 37.64% | 2.21% |
| Yingdong Zhang, 2001 [49] | 34.9% | 46.5% | 18.6% |
|  | 50.0% | 38.1% | 11.9% |
| Atsushi Araki, 2005 [50] | 35% | 47% | 18% |
| Mohammad A. Alam, 2007 [51] | 67.8% | 27.4% | 4.8% |
|  | 80% | 19% | 1% |
| Nadia Bouzidi, 2020 [52] | 59.5% | 37.8% | 2.7% |
|  | 42.2% | 38.9% | 18.9% |
| Guanzhong Ni, 2017 [53] | 50.54% | 43.01% | 6.45% |
| Jingwei Zhu, 2024 [54] | 28.9% | 55.6% | 15.6% |
|  | 44.6% | 49.1% | 6.3% |
| Xiaojie Yuan, 2019 [55] | 29.4% | 52.2% | 18.4% |
| Xue-bin Wang, 2015 [56] | 27.7% | 50.0% | 22.3% |
|  | 35.1% | 46.7% | 18.2% |
| CS Liu, 2007 [57] | 53.7% | 40.6% | 5.7% |
| Chien-Hsiung Cheng, 2008 [58] | 47.06% | 49.02% | 3.92% |
|  | 58.62% | 41.38% | 0.00% |
|  | 62.71% | 34.75% | 2.54% |
| Guancheng Yin, 2012 [59] | 41.2% | 43.2% | 15.5% |
|  | 38.9% | 35.7% | 25.5% |
| Saowanee Kajanachumpol, 2013 [60] | 64.2% | 32.1% | 3.7% |
|  | 61.6% | 34.9% | 3.5% |
| Hanaa H. Elsaid, 2020 [61] | 55.5% | 33% | 11.5% |
|  | 45% | 38% | 17% |
| Pethig Klaus, 1999 [62] | 38.36% | 52.74% | 8.90% |
| Gaetano Gorgone, 2012 [63] | 25% | 38.3% | 36.7% |
|  | 37.8% | 43.9% | 18.3% |
| Osvaldo P. Almeida, 2004 [64] | 12.08% | 47.08% | 40.83% |
| Chong Xie, 2019 [65] | 42.3% | 50% | 7.7% |
|  | 9.3% | 39.5% | 51.2% |
|  | 18.5% | 59.3% | 22.2% |
| Linjing Zhang, 2021 [66] | 45.56% | 42.22% | 12.22% |
|  | 72.22% | 20% | 7.78% |
| Xue Wu, 2022 [67] | 40.57% | 51.42% | 8.02% |
|  | 33.49% | 42.11% | 24.40% |
| Min Li, 2018 [68] | 25.00% | 53.85% | 21.15% |
|  | 32.43% | 50.00% | 17.57% |
| Yuelong Ji, 2014 [69] | 41.38% | 49.75% | 8.87% |
| Giuseppe Toffoli, 2003 [70] | 30.23% | 39.53% | 30.23% |
| Lijuan Ma, 2022 [71] | 15.9% | 29.6% | 54.5% |
|  | 37.8% | 49.4% | 12.8% |
| Domenico Dell'edera, 2013 [72] | 30.37% | 34.81% | 34.81% |
| Shanqun Jiang, 2012 [73] | 38.4% | 54.8% | 6.8% |
|  | 32.3% | 52.1% | 15.6% |
| Miroslav Petr, 2013 [74] | 40% | 50% | 10% |
| Zohreh Hekmati Azar Mehrabani, 2015 [75] | 53.3% | 26.7% | 20.0% |
| Vikki Ho, 2013 [76] | 41.3% | 44.4% | 14.3% |
| Shanqun Jiang, 2015 [77] | 25.96% | 49.04% | 25.00% |
|  | 34.65% | 37.80% | 27.56% |
| D Peterlana, 2005 [78] | 25.61% | 57.32% | 17.07% |
|  | 33.33% | 46.67% | 20.00% |
| Sebahattin Vurucu, 2008 [79] | 40.86% | 45.16% | 13.98% |
| Xiuwen Liang, 2022 [80] | 27.09% | 47.29% | 25.62% |
| Xueyun You, 2024 [81] | 54.26% | 41.70% | 4.04% |
|  | 49.66% | 39.60% | 10.74% |
| M.CANDITO, 1999 [82] | 38% | 40% | 22% |
| WenXing Li, 2017 [83] | 24.36% | 50.85% | 24.79% |
| Aline Barnabé, 2015 [84] | 47.3% | 41.5% | 11.2% |
| Kalman Benke, 2015 [85] | 87% | 10.3% | 2.7% |
|  | 89.2% | 10.8% | 0.0% |
|  | 88.2% | 11.8% | 0.0% |
|  | 40.7% | 37.8% | 22.2% |
|  | 71.4% | 28.6% | 0.0% |
|  | 7.6% | 46.2% | 46.2% |
| Nicoletta Botto, 2003 [86] | 29.4% | 58.8% | 11.8% |
|  | 27.5% | 49.0% | 23.5% |
| Ayse Feyda Nursal, 2021 [87] | 67.54% | 29.82% | 2.63% |
|  | 49.09% | 36.36% | 5.45% |
| Yan Zhang, 2024 [88] | 53.0% | 29.6% | 17.4% |
|  | 39.3% | 14.6% | 46.1% |
| K.Kostulas, 1998 [89] | 50% | 40% | 10% |
|  | 46% | 44% | 10% |
|  | 50% | 40% | 10% |
| Feijun Ye, 2022 [90] | 45.66% | 32.66% | 21.68% |
| Mark Lucock, 2000 [91] | 42% | 47% | 11% |
|  | 35% | 55% | 10% |
| Ryuichi Kawamoto, 2001 [92] | 41.72% | 41.72% | 16.56% |
| Ryuichi Kawamoto, 2004 [93] | 37.8% | 45.6% | 16.6% |
|  | 34.0% | 44.3% | 21.6% |
| Babak Saffari, 2013 [94] | 51.21% | 39.86% | 8.94% |
| Zhi Tang, 2017 [95] | 39.00% | 42.00% | 19.00% |
|  | 56.00% | 44.00% | 0.00% |
| I. Terruzzi, 2007 [96] | 26.9% | 63.5% | 9.6% |
|  | 21.4% | 64.3% | 14.3% |
| Monika Chorąży, 2019 [97] | 54% | 38.5% | 7.5% |
|  | 53.2% | 43% | 3.8% |
| Zhe Liang, 2024 [98] | 18.7% | 46.8% | 34.5% |
| Yan Wang, 2018 [99] | 17.9% | 44.9% | 37.2% |
|  | 14.7% | 52.2% | 47.2% |
|  | 13.1% | 47.4% | 39.4% |
|  | 14.3% | 49.0% | 36.7% |
|  | 14.1% | 49.8% | 36.1% |
| Souhir Chaabane, 2018 [100] | 24% | 59% | 17% |
|  | 47% | 47% | 6% |
| Sanjeev Kumar Pandey, 2013 [101] | 89.8% | 6.22% | 3.98% |
| Jun Luo, 2023 [102] | 24.6% | 52.3% | 23.1% |
|  | 37.0% | 46.9% | 16.1% |
| Binbin Wang, 2005 [103] | 48.1% | 36.5% | 15.4% |
|  | 60.8% | 36.2% | 3.0% |
| José Guillermo Buendía‑Pazarán, 2022 [104] | 21.7% | 45.6% | 32.6% |
|  | 34.8% | 39.1% | 26.1% |
| Chunquan Cai, 2019 [105] | 8.2% | 49.2% | 42.6% |
|  | 24.6% | 44.3% | 31.1% |
| Shan Liu, 2019 [106] | 21.4% | 47.0% | 31.6% |
|  | 19.9% | 47.0% | 33.1% |
| Iman K. Al-Absi, 2006 [107] | 74.5% | 19.3% | 6.2% |
|  | 65.3% | 30.6% | 4.1% |
| Li Chen, 2025 [108] | 25.56% | 47.78% | 26.67% |
|  | 44.30% | 43.04% | 12.66% |
| Boyi Yang, 2021 [109] | 36.4% | 42.0% | 21.6% |
| Carlos L. Krumdieck, 2008 [110] | 59.79% | 25.77% | 14.43% |
| Federica Boraldi, 2014 [111] | 34.0% | 48.5% | 17.5% |
|  | 24.3% | 48.0% | 27.7% |
| Liuqiang Huang, 2020 [112] | 66.49% | 27.57% | 5.95% |
| Mei Luo, 2015 [113] | 48% | 40% | 12% |
|  | 51% | 43% | 6% |
| L Torres-Sa´nchez, 2006 [114] | 21.5% | 52.3% | 26.2% |
| Qiannan Guo, 2022 [115] | 13.66% | 47.72% | 38.62% |
|  | 17.14% | 52.86% | 30.00% |
|  | 35.42% | 42.71% | 21.88% |
| Anzel Bahadır, 2014 [116] | 29.0% | 57.9% | 13.1% |
| Alexandre C. Pereira, 2007 [117] | 44.8% | 43.4% | 11.8% |
| Song Zhang, 2021 [118] | 12.7% | 30.91% | 56.36% |
|  | 16.36% | 25.45% | 58.18% |
| Crystal Yin Tung Chan, 2017 [119] | 60% | 30% | 10% |
|  | 51.61% | 38.71% | 9.68% |
| Cinzia Fatini, 2005 [120] | 24.6% | 51.3% | 24.1% |
| Ningyuan Chen, 2014 [121] | 69.38% | 26.36% | 4.26% |
| Wenxing Li, 2016 [122] | 24.6% | 50.4% | 25.0% |
| Tarciana Guedes, 2022 [123] | 44.26% | 50.28% | 4.92% |
|  | 39.56% | 49.45% | 10.99% |
| Xiaowei Ng, 2008 [124] | 45.76% | 38.98% | 15.25% |
| Inmaculada Martín, 2003 [125] | 46.58% | 34.93% | 18.49% |
| Andrew G. Bostom, 1996 [126] | 43% | 44% | 13% |
| Cornelie Nienaber-Rousseau, 2013 [127] | 84% | 15.2% | 0.8% |
| Guangsen Zhang, 2001 [128] | 37.0% | 47.0% | 16.0% |
|  | 41.1% | 45.7% | 13.2% |
| Carolyn M. Summers, 2010 [129] | 69.6% | 30.4% | 0.0% |
|  | 30.8% | 50.0% | 19.2% |
|  | 38.1% | 61.9% | 0.0% |
| Rossella Marcucci, 2003 [130] | 10.9% | 50.9% | 38.1% |
|  | 29.5% | 55.7% | 14.7% |
| JX Chen, 2014 [131] | 42.2% | 47.1% | 10.7% |
|  | 36.3% | 47.7% | 16.0% |
| J.M. Biselli, 2008 [132] | 40.3% | 48.6% | 11.1% |
|  | 51.5% | 39.7% | 8.8% |
| J. M. Biselli, 2012 [133] | 45.6% | 41.1% | 13.3% |
| Binghui Du, 2018 [134] | 22.15% | 44.00% | 33.85% |
|  | 10.86% | 41.85% | 47.29% |
| Santiago G. Miriuka, 2004 [135] | 46.4% | 39.2% | 14.2% |
|  | 48.6% | 41.5% | 9.8% |
| Grazyna Gromadzka, 2011 [136] | 40.0% | 53.5% | 6.5% |
| Ali Erkan Aşcı, 2024 [137] | 57.1% | 30.2% | 12.7% |
|  | 57.8% | 34.7% | 7.5% |
| Boyi Yang, 2013 [138] | 32.84% | 43.92% | 23.24% |
| Wei Zheng, 2024 [139] | 20.41% | 47.30% | 32.29% |
| Jacomina P. du Plessis, 2022 [140] | 84.0% | 15.2% | 0.80% |
| Francesco Sofi, 2005 [141] | 32.2% | 49.5% | 18.3% |
|  | 37.9% | 48.2% | 13.9% |
| L. Flicker, 2004 [142] | 41.8% | 46.5% | 11.7% |
| Maria C. Rodriguez-Oroz, 2009 [143] | 32.1% | 46.5% | 21.4% |
|  | 42.9% | 53.2% | 3.9% |
| Alexandre Rodrigues Guerzoni, 2008 [144] | 36.55% | 55.17% | 8.28% |
| Dindagur Nagaraja, 2008 [145] | 85.2% | 14.8% | 0.0% |
|  | 85.7% | 12.7% | 1.6% |
| Richard M. Camicioli, MD, 2009 [146] | 39.22% | 50.98% | 9.80% |
|  | 34.69% | 46.94% | 18.37% |
| Francesco Orio, JR, 2003 [147] | 22.9% | 58.5% | 18.6% |
|  | 24.3% | 54.3% | 21.4% |
| Bruno Zappacosta, 2013 [148] | 42.9% | 31.4% | 25.7% |
|  | 33.3% | 38.5% | 28.2% |
|  | 32.4% | 40.5% | 27.1% |
|  | 34.2% | 39.5% | 26.3% |
| Helena Caldeira-Araújo, 2019 [149] | 52.2% | 38.6% | 9.2% |
| Wolfgang Herrmann, 2003 [150] | 46% | 43% | 11% |
|  | 44% | 42% | 14% |
|  | 34% | 47% | 19% |
| T. Bjerregaard Larsen, 2002 [151] | 39.6% | 45.3% | 15.1% |
|  | 50.3% | 41.4% | 8.3% |
| J.A. He, 2010 [152] | 24% | 43% | 33% |
|  | 35% | 48% | 17% |
| Hui Cao, 2014 [153] | 29.8% | 50.8% | 19.4% |
|  | 33.7% | 54.4% | 11.9% |
| Gianluca Sottilotta, 2010 [154] | 34% | 38% | 28% |
|  | 25% | 44% | 31% |
| Maria Rosa Biagini, 2006 [155] | 27.45% | 41.18% | 31.37% |
| Venkata Pinnelli Bharatkumar, 2012 [156] | 80.5% | 15.7% | 3.8% |
|  | 84.7% | 13.3% | 2.2% |
| Paolo Ventura, 2016 [157] | 72.00% | 22.00% | 6.00% |
|  | 74.19% | 17.74% | 8.06% |
|  | 31.58% | 34.21% | 34.21% |
| Alfredo Papa, 2001 [158] | 32.8% | 50% | 17.2% |
|  | 44.6% | 37.4% | 17.9% |
| Wolfgang Herrmann, 2003 [159] | 35% | 47% | 18% |
|  | 45% | 42% | 12% |
| Olfa Ben Salem-Berrabah, 2010 [160] | 66% | 30% | 4% |
|  | 58.76% | 36.08% | 5.15% |
| Vincenzo Belcastro, 2010 [161] | 26.25% | 45.95% | 27.80% |
|  | 25.97% | 47.62% | 26.41% |
| Zoia Rossokha, 2022 [162] | 43.69% | 44.66% | 11.65% |
| Gaetano Gorgone, 2009 [163] | 31.03% | 44.83% | 24.14% |
|  | 23.33% | 50.00% | 26.67% |
| Eduarda Bonelli Zarur, 2025 [164] | 47.2% | 44.4% | 8.3% |
|  | 37.7% | 52.2% | 10.1% |
| Gaetano Gorgone, 2009 [165] | 28.6% | 39.7% | 31.7% |
|  | 41.3% | 44.4% | 15.9% |
| J.R. Faria-Neto, 2006 [166] | 60% | 30.9% | 9.1% |
| Mai A. Abd-Elmawla, 2016 [167] | 73% | 15% | 12% |
|  | 89% | 8% | 3% |
| Anna Waśkiewicz, 2011 [168] | 48.03% | 42.22% | 9.75% |
| Tsu-Shiu Hsu, 2001 [169] | 57.83% | 33.73% | 8.43% |
|  | 54.81% | 39.42% | 5.77% |
| Ewa Strauss, 2003 [170] | 65% | 27% | 8% |
|  | 33% | 60% | 6% |
| Tarek A. Abd El-Aziz, 2017 [171] | 49.4% | 35.6% | 15.0% |
|  | 56.7% | 37.5% | 5.8% |
| Qiang Zeng, 2016 [172] | 22.52% | 49.08% | 28.40% |
| Aissam El Maataoui, 2022 [173] | 64.3% | 28.6% | 7.1% |
|  | 51.65% | 42.3% | 6.05% |
| Zoe Yates, 2003 [174] | 38.0% | 44.0% | 18.0% |
|  | 40.4% | 45.5% | 14.1% |
| Emilia Balogh, 2012 [175] | 45.36% | 44.04% | 10.60% |
|  | 44.12% | 50.00% | 5.88% |
|  | 40.38% | 47.75% | 11.87% |
|  | 43.97% | 47.56% | 8.47% |
| A Pezzini, 2006 [176] | 29.3% | 49.4% | 21.3% |
|  | 38.7% | 47.1% | 14.2% |
| F.M. Amaral, 2017 [177] | 50.59% | 44.71% | 4.71% |
| IO Oliveira, 2017 [178] | 48.04% | 42.78% | 9.18% |
| Javed Y. Fowdar, 2012 [179] | 45.1% | 46.2% | 8.8% |
|  | 44.5% | 46.6% | 8.9% |
| Ewa Strauss, 2017 [180] | 49.0% | 41.7% | 9.4% |
|  | 36.4% | 47.3% | 16.4% |
|  | 46.52% | 43.82% | 9.66% |
| Alfredo Mazza, 2000 [181] | 36.15% | 40.77% | 23.08% |
| Nahid Yazdanpanah, 2008 [182] | 45.28% | 44.39% | 10.33% |
| Priyanka Kumari, 2013 [183] | 70.0% | 26.8% | 3.2% |
|  | 77.6% | 21.3% | 1.1% |
| Ángel José González Ordóñez, 2000 [184] | 38.78% | 53.06% | 8.16% |
| Hui Rao, 2022 [185] | 55.19% | 38.28% | 6.53% |
|  | 56.29% | 37.46% | 6.24% |
| Marı´a L. Iglesias Varela, 2001 [186] | 28.99% | 55.80% | 15.22% |
|  | 34.03% | 50.69% | 15.28% |
| Teˆmis Maria Fe´ lix, 2004 [187] | 46.34% | 39.02% | 14.63% |
|  | 47.72% | 36.36% | 15.90% |
|  | 46.34% | 36.58% | 17.07% |
|  | 36.36% | 50.0% | 13.36% |
| V.O. Osunkalu, 2020 [188] | 76.6% | 22.3% | 1.0% |
|  | 59.8% | 31.2% | 9.0% |
| M. Messedi, 2013 [189] | 40.1% | 45.1% | 14.8% |
|  | 45.3% | 45.9% | 8.7% |
| Guancheng Yin, 2012 [190] | 37.6% | 48.1% | 14.3% |
|  | 33.5% | 45.1% | 21.4% |
| Najiba Fekih-Mrissa, 2017 [191] | 35.0% | 63.75% | 1.25% |
|  | 62% | 34% | 4% |
| Mohammad Soleiman Soltanpour, 2013 [192] | 60.3% | 27.4% | 12.3% |
|  | 61.6% | 34.3% | 4.1% |
| Shanqun Jiang, 2016 [193] | 37.7% | 50.9% | 11.3% |
|  | 34.0% | 49.1% | 17.0% |
|  | 33.8% | 54.9% | 11.3% |
|  | 33.3% | 47.8% | 18.8% |
| Mohit Kumar, 2018 [194] | 78% | 20% | 2% |
|  | 32% | 53% | 15% |
| Muammer Karadeniz, 2010 [195] | 17.4% | 75.6% | 7% |
|  | 50% | 40% | 10% |
| Xin Fang, 2005 [196] | 31% | 43% | 26% |
|  | 15% | 60% | 25% |
|  | 67% | 25% | 8% |
| M.R. Safarinejad, 2011 [197] | 30.3% | 53.9% | 15.8% |
|  | 44.1% | 44.4% | 11.5% |
| Renuka Munshi, 2019 [198] | 86.67% | 12.50% | 0.83% |
|  | 74.58% | 22.50% | 2.92% |
| Lei Chen, 2023 [199] | 15.7% | 45.0% | 39.3% |
|  | 22.0% | 53.3% | 24.7% |
| Takamichi Nakamura, 2002 [200] | 33.58% | 51.03% | 15.38% |
| Ana Beatriz Alvarez Perez, 2003 [201] | 45.03% | 47.33% | 7.64% |
|  | 51.13% | 42% | 6.87% |
|  | 53.97% | 35.71% | 10.32% |
|  | 55.56% | 42.85% | 1.59% |
| Domenico Girelli, 2013 [202] | 30.66% | 51.09% | 18.25% |
|  | 32.4% | 53.2% | 14.4% |
| M Erol Turaçli, 2005 [203] | 51.3% | 40.8% | 7.9% |
|  | 52.9% | 35.3% | 11.8% |
| Maria Grazia Andreassi, 2003 [204] | 28.26% | 47.83% | 23.91% |
| Ercan Karabacak, 2014 [205] | 43.33% | 43.33% | 13.33% |
| Rey-Yue Yuan, 2019 [206] | 43.8% | 45.8% | 10.4% |
|  | 35.7% | 53.6% | 10.7% |
|  | 59.1% | 34.5% | 6.4% |
| Zhanqi Feng, 2024 [207] | 24.3% | 59.0% | 16.6% |
|  | 4.9% | 24.1% | 71.1% |
| J. Kalita, 2006 [208] | 67.24% | 27.59% | 5.17% |
| Erika RF Siqueira, 2011 [209] | 53.8% | 40.4% | 5.7% |
|  | 51.4% | 41.4% | 7.1% |
|  | 56.9% | 37.9% | 5.1% |
|  | 44.8% | 46.5% | 8.6% |
|  | 60.1% | 35.4% | 4.4% |
|  | 39.3% | 50.8% | 9.8% |
| Tao Huang, 2011 [210] | 43.20% | 44.62% | 12.18% |
| Najiba Fekih-Mrissa, 2013 [211] | 41.6% | 51.2% | 7.15% |
|  | 60% | 35% | 5% |
| Maria José Franco Brochado, 2013 [212] | 43% | 42% | 15% |
|  | 37% | 50% | 13% |
| Antonio Arauz, 2007 [213] | 20.51% | 69.23% | 10.26% |
|  | 28.95% | 65.79% | 5.26% |
| Nasim Mansoori, 2012 [214] | 63.7% | 32.5% | 3.8% |
|  | 70.0% | 28.0% | 2.0% |
|  | 74.2% | 24.2% | 1.6% |
| Guilherme da Silva Silvestre, 2023 [215] | 47.8% | 42.5% | 9.7% |
|  | 48.4% | 36.3% | 15.3% |
| Aruna Poduri, 2008 [216] | 73.84% | 26.16% | 0.00% |
|  | 82.52% | 17.48% | 0.00% |
| Haidy E. Zidan, 2013 [217] | 38.8% | 31.2% | 30% |
|  | 26.3% | 37.5% | 36.2% |
| Miaonan Li, 2017 [218] | 25.16% | 44.19% | 30.65% |
| Zhuoran Li, 2022 [219] | 41.78% | 38.82% | 19.41% |
| Ayse Feyda Nursal, 2017 [220] | 48.0% | 42.4% | 9.6% |
|  | 66.0% | 31.0% | 3.0% |
| Linjing Zhang, 2021 [221] | 4.88% | 58.54% | 36.59% |
|  | 32.93% | 60.98% | 6.10% |
| iPatrícia Matos Biselli, 2009 [222] | 35% | 54% | 11% |
|  | 36% | 55% | 9% |
| Aleksandra Sniezawska, 2011 [223] | 44% | 52% | 4% |
|  | 55% | 34% | 11% |
| Jianhao Guo, 2022 [224] | 25.93% | 53.33% | 20.74% |
| Manuela Fodinger, 1997 [225] | 42.6% | 43.4% | 14.0% |
|  | 39.1% | 50.7% | 10.2% |
| Jimmy Crott, 2001 [226] | 42.31% | 38.46% | 19.23% |
| Mirande Candito, 2008 [227] | 32.5% | 51.9% | 15.6% |
|  | 42.6% | 47.5% | 9.9% |
| Jolanta Dorszewska, 2007 [228] | 55% | 40% | 5% |
|  | 38% | 53% | 9% |
|  | 49% | 43% | 8% |
| Natalia Ubeda, 2011 [229] | 35.7% | 57.2% | 7.1% |
| C Mislanova, 2011 [230] | 42.8% | 39.3% | 17.8% |
| Huipu Xu, 2013 [231] | 22.4% | 44.9% | 32.7% |
|  | 45.7% | 42.8% | 11.4% |
| Fausi Rassoul, 2000 [232] | 56% | 34% | 10% |
|  | 50% | 36.7% | 13.3% |
| Montserrat Creus, 2013 [233] | 38.3% | 43.3% | 18.3% |
|  | 43.3% | 43.3% | 13.3% |
| Yuan YG, 2008 [234] | 39.7% | 41.4% | 19.0% |
|  | 33.8% | 47.5% | 18.8% |
| Giuseppe Minniti, 2014 [235] | 30% | 49% | 21% |
|  | 42% | 38% | 20% |
| Noomen Bouaziz, 2010 [236] | 76% | 20% | 4% |
|  | 72% | 16% | 12% |
| Akash Nahar, 2011[237] | 56.03% | 39.72% | 4.26% |
| Angela Passaro, 2001 [238] | 23.33% | 60.00% | 16.67% |
| Sylene Coutinho Rampche de Carvalho, 2013 [239] | 34.29% | 48.57% | 17.14% |
| M. Palep-Singh, 2007 [240] | 61.97% | 33.80% | 4.23% |
| Paik-Seong Lim, 2001 [241] | 58% | 35.4% | 6.6% |
|  | 58% | 38% | 4% |
| Adriano Sabino, 2009 [242] | 64.9% | 32.4% | 2.7% |
|  | 53.5% | 41.9% | 4.6% |
|  | 57.1% | 42.9% | 0.0% |
|  | 34.1% | 52.3% | 13.6% |
| Junli Song, 2020 [243] | 19.59% | 49.95% | 30.46% |
| Jiazhong Sun, 2009 [244] | 54.9% | 26.8% | 18.3% |
|  | 43.7% | 34.0% | 22.3% |
| Eulo Lupi-Herrera, 2018 [245] | 36.3% | 50% | 13.7% |
|  | 27% | 44.3% | 28.7% |
|  | 26.3% | 57.9% | 15.8% |
|  | 35.6% | 43.9% | 20.5% |
|  | 27.9% | 61.7% | 10.3% |
|  | 34.3% | 44.4% | 21.3% |
|  | 18.7% | 56.2% | 25% |
|  | 33.6% | 47.5% | 18.9% |
| Ana Claudia M. Ale´ssio, 2004 [246] | 46.5% | 44% | 9.5% |
| B Akoglu, 2008 [247] | 33% | 48% | 19% |
| Peter Chedraui, 2014 [248] | 39.3% | 48.7% | 12.0% |
|  | 31.3% | 60.7% | 8.0% |
| Eva Mariane Mantjoro, 2016 [249] | 43.2% | 45.3% | 11.6% |
|  | 47.5% | 40.8% | 11.8% |
| Hyunjung Kim, 2016 [250] | 29% | 45.9% | 25.1% |
| Yayun Xu, 2024 [251] | 49.78% | 38.05% | 12.17% |
| Salwa Ibrahim, 2009 [252] | 60% | 32% | 8% |
| Yesim Ozarda, 2009 [253] | 49.3% | 9.7% | 41.0% |
| Arve Ulvik, 2017 [254] | 49.6% | 42.0% | 8.4% |
| Chi Zhang, 2024 [255] | 5.11% | 22.63% | 72.26% |
|  | 22.54% | 53.52% | 23.94% |
| Oushan Tang, 2014 [256] | 30% | 54% | 16% |
|  | 56% | 36% | 8% |
| Xijiang Hu, 2023 [257] | 50.40% | 38.26% | 11.35% |
|  | 52.56% | 37.18% | 10.26% |
|  | 30.30% | 46.97% | 22.73% |
| Yingdong Zhang, 2005 [258] | 33.33% | 48.04% | 18.63% |
|  | 37.14% | 42.86% | 20.00% |
| K.K. Sukla, 2015 [259] | 60.26% | 37.09% | 2.65% |
|  | 56.95% | 39.07% | 3.97% |
|  | 54.97% | 38.41% | 6.62% |
|  | 74.50% | 23.50% | 2.00% |
|  | 75.81% | 22.58% | 1.61% |
|  | 77.00% | 21.00% | 2.00% |
| Giangennaro Coppola, 2012 [260] | 35.90% | 46.15% | 17.95% |
| Santosh Kumar Gupta, 2012 [261] | 66.3% | 32.2% | 1.5% |
|  | 77.5% | 22.0% | 0.5% |
| Mohammad Reza Safarinejad, 2010 [262] | 35.1% | 49.1% | 15.8% |
|  | 44.7% | 43.9% | 11.4% |
| Yunguo Wang, 2023 [263] | 19.14% | 48.64% | 32.22% |
| Ali Yasar, 2012 [264] | 62.5% | 32.5% | 5% |
|  | 50% | 37.5% | 12.5% |
| Viktor Komlósi, 2010 [265] | 47% | 40% | 13% |
|  | 44% | 42% | 14% |
|  | 47% | 41% | 12% |
|  | 40% | 48% | 12% |
| Hongyu Yuan, 2020 [266] | 17.18% | 42.33% | 40.49% |
|  | 32.52% | 34.66% | 32.82% |
| Juan Ni, 2018 [267] | 44.3% | 45.0% | 15.7% |
| Albino Carrizzo, 2022 [268] | 27.03% | 37.84% | 35.14% |
| Hui Shi, 2020 [269] | 30.72% | 51.96% | 17.32% |
|  | 31.95% | 47.63% | 20.41% |
| Laila Rashed, 2017 [270] | 53.3% | 43.3% | 3.3% |
|  | 47.3% | 38.2% | 14.5% |
| C Wilmanns, 2011 [271] | 34% | 41% | 24% |
|  | 58% | 38% | 4% |
| Liang Ma, 2017 [272] | 15.25% | 44.92% | 39.83% |
|  | 22.56% | 46.92% | 30.51% |
| Tianyuan Xiang, 2020 [273] | 23.06% | 48.63% | 28.31% |
| Nevin Ilhan, 2008 [274] | 52.0% | 44.0% | 4.0% |
|  | 46.2% | 41.0% | 12.8% |
| Nadia Ferlazzo, 2011 [275] | 24.6% | 46.4% | 29% |
|  | 37.7% | 47.8% | 14.5% |
| Chuanfei Chen, 2010 [276] | 51.3% | 42.5% | 6.2% |
|  | 75.6% | 24.4% | 0.0% |
|  | 80.1% | 18.6% | 1.3% |
| Zhe Liang, 2024 [277] | 18.5% | 46.7% | 34.8% |
| Baeck Hee Lee, 2000 [278] | 47% | 41% | 12% |
|  | 26% | 58% | 16% |
| Shuang Liang, 2014 [279] | 34.20% | 44.30% | 21.50% |
| Nurten Dinç, 2016 [280] | 46.88% | 41.67% | 11.46% |
| Jinghong Rao, 2023 [281] | 25.63% | 47.50% | 26.88% |
|  | 23.13% | 32.50% | 44.38% |
| Chia-Lun Chao, 1999 [282] | 53% | 37% | 10% |
|  | 52% | 40% | 8% |
|  | 54% | 33% | 13% |
| Chao-Hung Ho, 2000 [283] | 47.7% | 36.9% | 15.5% |
| Sung Eun Cho, 2006 [284] | 29.0% | 44.1% | 26.9% |
| Izabela Nowak, 2017 [285] | 39.76% | 51.00% | 9.24% |
|  | 52.50% | 42.50% | 5.00% |
| Andrea Stoccoro, 2017 [286] | 28.6% | 49.0% | 22.4% |
|  | 34.8% | 45.5% | 19.7% |
| Xiaolin Wang, 2019 [287] | 37.04% | 48.56% | 14.4% |
|  | 35.57% | 52.58% | 11.86% |
| Mizuya Fukasawa, 2003 [288] | 32.94% | 46.88% | 20.18% |
| S.J. Carlus, 2016 [289] | 70.8% | 25.4% | 3.8% |
|  | 70.3% | 23.1% | 6.6% |
|  | 76.7% | 23.3% | 0.0% |
|  | 62.8% | 23.3% | 14.0% |
| Annamaria Vallelunga, 2013 [290] | 28.10% | 49.59% | 22.31% |
| M. P. Hermans, 2005 [291] | 44% | 41% | 15% |
|  | 17% | 57% | 26% |
| J.M. Biselli, 2008 [292] | 41.07% | 42.86% | 16.07% |
| Xiaolin Qian, 2021 [293] | 36.1% | 46.0% | 17.9% |
| Soh Murakami, 2001 [294] | 34.3% | 49.0% | 16.7% |
| Elene Asanidze, 2025 [295] | 35.7% | 50.8% | 13.5% |
|  | 73.1% | 18.9% | 8% |
|  | 61.3% | 25.9% | 12.8% |
|  | 80.9% | 16.7% | 2.4% |
| F Wang, 2019 [296] | 9.22% | 43.20% | 47.58% |
|  | 20.77% | 57.31% | 21.92% |
| Xinlei Mao, 2018 [297] | 41.41% | 50.51% | 8.08% |
|  | 26.92% | 60.77% | 12.31% |
|  | 23.90% | 69.18% | 6.92% |
| Robert Loncar, 2006 [298] | 40% | 48% | 11.4% |
|  | 44% | 47% | 8.3% |
| R. Marcucci, F. Sofi, 2004 [299] | 31.87% | 46.50% | 21.63% |
|  | 37.72% | 46.50% | 15.77% |

**References**

1. Zhao X, Yang L, Wang M, Zhang X. Correlation of Folic Acid Metabolic Gene Polymorphisms, Homocysteine, Vitamin B12, and Red Blood Cell Folate with Adverse Pregnancy Outcomes. Altern Ther Health Med. 2024 May;30(5):284-288.
2. Ravaglia G, Forti P, Maioli F, Scali RC, Arnone G, Talerico T, Pantieri T, Nativio V, Mantovani V, Bianchin M. Common polymorphisms in methylenetetrahydrofolate reductase (MTHFR): relationships with plasma homocysteine concentrations and cognitive status in elderly northern italian subjects. Arch Gerontol Geriatr Suppl. 2004;(9):339-48. doi: 10.1016/j.archger.
3. Ferroni P, Palmirotta R, Martini F, Riondino S, Savonarola A, Spila A, Ciatti F, Sini V, Mariotti S, Del Monte G, Roselli M, Guadagni F. Determinants of homocysteine levels in colorectal and breast cancer patients. Anticancer Res. 2009 Oct;29(10):4131-8.
4. Kalita J, Singh VK, Misra UK. A study of hyperhomocysteinemia in cerebral venous sinus thrombosis. Indian J Med Res. 2020 Dec;152(6):584-594. doi: 10.4103/ijmr.IJMR_2125_18.
5. Wen C, Lv JF, Wang L, Zhu WF, Wan FS, Wang XZ. Association of a methylene tetrahydrofolate reductase C677T polymorphism with several blood chemical levels in a Chinese population. Genet Test Mol Biomarkers. 2015 Jan;19(1):24-9. doi: 10.1089/gtmb.2014.0213.
6. Paul S, Sadhukhan S, Munian D, Bankura B, Das M. Association of FOLH1, DHFR, and MTHFR gene polymorphisms with susceptibility of Neural Tube Defects: A case control study from Eastern India. Birth Defects Res. 2018 Aug 15;110(14):1129-1138. doi: 10.1002/bdr2.1365.
7. Zhang J, Zeng C, Huang X, Liao Q, Chen H, Liu F, Sun D, Luo S, Xiao Y, Xu W, Zeng D, Song M, Tian F. Association of homocysteine and polymorphism of methylenetetrahydrofolate reductase with early-onset post stroke depression. Front Nutr. 2022 Dec 6;9:1078281. doi: 10.3389/fnut.2022.1078281.
8. Lioudyno VI, Tsymbalova EA, Chernyavskaya EA, Scripchenko EY, Bisaga GN, Dmitriev AV, Abdurasulova IN. Association of Increased Homocysteine Levels with Impaired Folate Metabolism and Vitamin B Deficiency in Early-Onset Multiple Sclerosis. Biochemistry (Mosc). 2024 Mar;89(3):562-573. doi: 10.1134/S0006297924030143.
9. Zuntar I, Antoljak N, Vrkić N, Topić E, Kujundzić N, Demarin V, Vuković V. Association of methylenetetrahydrofolate (MTHFR) and apolipoprotein E (apo E) genotypes with homocysteine, vitamin and lipid levels in carotid stenosis. Coll Antropol. 2006 Dec;30(4):871-8.
10. Sukla KK, Raman R. Association of MTHFR and RFC1 gene polymorphism with hyperhomocysteinemia and its modulation by vitamin B12 and folic acid in an Indian population. Eur J Clin Nutr. 2012 Jan;66(1):111-8. doi: 10.1038/ejcn.2011.152.
11. Koubaa N, Nakbi A, Hammami S, Mehri S, Attia N, Ben Farhat M, Hammami M. Association of the C677T MTHFR polymorphism with homocysteine, ox-LDL levels, and thiolactonase activities in the severity of coronary syndrome. Clin Appl Thromb Hemost. 2010 Oct;16(5):515-21. doi: 10.1177/1076029610369798.
12. Huang LW, Li LL, Li J, Chen XR, Yu M. Association of the methylenetetrahydrofolate reductase (*MTHFR*) gene variant C677T with serum homocysteine levels and the severity of ischaemic stroke: a case-control study in the southwest of China. J Int Med Res. 2022 Feb;50(2):3000605221081632. doi: 10.1177/03000605221081632.
13. Dhillon VS, Shahid M, Husain SA. Associations of MTHFR DNMT3b 4977 bp deletion in mtDNA and GSTM1 deletion, and aberrant CpG island hypermethylation of GSTM1 in non-obstructive infertility in Indian men. Mol Hum Reprod. 2007 Apr;13(4):213-22. doi: 10.1093/molehr/gal118.
14. Guan D, Ji Y, Lu X, Feng W, Ma W. Associations of MTHFR gene polymorphism with lipid metabolism and risk of cerebral infarction in the Northwest Han Chinese population. Front Neurol. 2023 Mar 31;14:1152351. doi: 10.3389/fneur.2023.1152351.
15. Somekawa Y, Kobayashi K, Tomura S, Aso T, Hamaguchi H. Effects of hormone replacement therapy and methylenetetrahydrofolate reductase polymorphism on plasma folate and homocysteine levels in postmenopausal Japanese women. Fertil Steril. 2002 Mar;77(3):481-6. doi: 10.1016/s0015-0282(01)03228-9.
16. Guo H, Song R. Factors associated with hyperhomocysteinemia in relatively healthy adult males in northern Shaanxi, China: A cross-sectional study. Medicine (Baltimore). 2025 May 2;104(18):e42377. doi: 10.1097/MD.0000000000042377.
17. Coppedè F, Tannorella P, Pezzini I, Migheli F, Ricci G, Caldarazzo lenco E, Piaceri I, Polini A, Nacmias B, Monzani F, Sorbi S, Siciliano G, Migliore L. Folate, homocysteine, vitamin B12, and polymorphisms of genes participating in one-carbon metabolism in late-onset Alzheimer's disease patients and healthy controls. Antioxid Redox Signal. 2012 Jul 15;17(2):195-204. doi: 10.1089/ars.2011.4368.
18. Jiajin L, Shuyan C, Ying W, Junxiao C, Xiudi W. Genetic polymorphisms in folate metabolism as risk for Down syndrome in the southern China. J Matern Fetal Neonatal Med. 2019 Jun;32(12):2030-2035. doi: 10.1080/14767058.2018.1424818.
19. Senemar S, Saffari B, Sharifkazemi MB, Bahari M, Jooyan N, Dehaghani ED, Yavarian M. 5,10-methylene tetrahydrofolate reductase C677T gene polymorphism, homocysteine concentration and the extent of premature coronary artery disease in southern Iran. EXCLI J. 2013 May 16;12:437-48.
20. Zappacosta B, Graziano M, Persichilli S, Di Castelnuovo A, Mastroiacovo P, Iacoviello L. 5,10-Methylenetetrahydrofolate reductase (MTHFR) C677T and A1298C polymorphisms: genotype frequency and association with homocysteine and folate levels in middle-southern Italian adults. Cell Biochem Funct. 2014 Jan;32(1):1-4. doi: 10.1002/cbf.3019.
21. Clément A, Menezo Y, Cohen M, Cornet D, Clément P. 5-Methyltetrahydrofolate reduces blood homocysteine level significantly in C677T methyltetrahydrofolate reductase single-nucleotide polymorphism carriers consulting for infertility. J Gynecol Obstet Hum Reprod. 2020 Jan;49(1):101622. doi: 10.1016/j.jogoh.2019.08.005.
22. Giusti B, Gori AM, Marcucci R, Sestini I, Saracini C, Sticchi E, Gensini F, Fatini C, Abbate R, Gensini GF. Role of C677T and A1298C MTHFR, A2756G MTR and -786 C/T eNOS gene polymorphisms in atrial fibrillation susceptibility. PLoS One. 2007 Jun 6;2(6):e495. doi: 10.1371/journal.pone.0000495.
23. Ozkul Y, Evereklioglu C, Borlu M, Taheri S, Calis M, Dündar M, Ilhan O. 5,10-Methylenetetrahydrofolate reductase C677T gene polymorphism in Behcet's patients with or without ocular involvement. Br J Ophthalmol. 2005 Dec;89(12):1634-7. doi: 10.1136/bjo.2005.076836.
24. Kimura H, Gejyo F, Suzuki S, Takeda T, Miyazaki R, Yoshida H. A C677T mutation in the methylenetetrahydrofolate reductase gene modifies serum cysteine in dialysis patients. Am J Kidney Dis. 2000 Nov;36(5):925-33. doi: 10.1053/ajkd.2000.19085.
25. Long Y, Zhao XT, Liu C, Sun YY, Ma YT, Liu XY, Liu JX. A Case-Control Study of the Association of the Polymorphisms of MTHFR and APOE with Risk Factors and the Severity of Coronary Artery Disease. Cardiology. 2019;142(3):149-157. doi: 10.1159/000499866.
26. Li A, Shi Y, Xu L, Zhang Y, Zhao H, Li Q, Zhao X, Cao X, Zheng H, He Y. A possible synergistic effect of MTHFR C677T polymorphism on homocysteine level variations increased risk for ischemic stroke. Medicine (Baltimore). 2017 Dec;96(51):e9300. doi: 10.1097/MD.0000000000009300.
27. Du X, Xiao L, Sun R, Li K, Liang L, Song L, Liu Z. A prospective cohort study of MTHFR C677T gene polymorphism and its influence on the therapeutic effect of homocysteine in stroke patients with hyperhomocysteinemia. BMC Neurol. 2020 Apr 11;20(1):128. doi: 10.1186/s12883-020-01701-8.
28. van der Put NM, Gabreëls F, Stevens EM, Smeitink JA, Trijbels FJ, Eskes TK, van den Heuvel LP, Blom HJ. A second common mutation in the methylenetetrahydrofolate reductase gene: an additional risk factor for neural-tube defects? Am J Hum Genet. 1998 May;62(5):1044-51. doi: 10.1086/301825.
29. da Silva VC, Ramos FJ, Freitas EM, de Brito-Marques PR, Cavalcanti MN, D'Almeida V, Cabral-Filho JE, Muniz MT. Alzheimer's disease in Brazilian elderly has a relation with homocysteine but not with MTHFR polymorphisms. Arq Neuropsiquiatr. 2006 Dec;64(4):941-5. doi: 10.1590/s0004-282x2006000600010.
30. Dedoussis GV, Panagiotakos DB, Pitsavos C, Chrysohoou C, Skoumas J, Choumerianou D, Stefanadis C; ATTICA Study Group. An association between the methylenetetrahydrofolate reductase (MTHFR) C677T mutation and inflammation markers related to cardiovascular disease. Int J Cardiol. 2005 Apr 28;100(3):409-14. doi: 10.1016/j.ijcard.2004.08.038.
31. Liu Y, Pu G, Yang C, Wang Y, Jin K, Wang S, Liang X, Hu S, Sun S, Lai M. Association analysis of MTHFR (rs1801133 and rs1801131) gene polymorphism towards the development of type 2 diabetes mellitus in Dali area population from Yunnan Province, China. PeerJ. 2024 Oct 24;12:e18334. doi: 10.7717/peerj.18334.
32. Cai W, Yin L, Yang F, Zhang L, Cheng J. Association between Hcy levels and the *CBS844ins68* and *MTHFR C677T* polymorphisms with essential hypertension. Biomed Rep. 2014 Nov;2(6):861-868. doi: 10.3892/br.2014.357.
33. Sun C, Ding D, Wen Z, Zhang C, Kong J. Association between Micronutrients and Hyperhomocysteinemia: A Case-Control Study in Northeast China. Nutrients. 2023 Apr 14;15(8):1895.
34. Li ZC, Huang M, Yao QY, Lin CH, Hong BC, Wang JH, Zhang Z. Association between *MTHFR C677T* Gene Polymorphisms and the Efficacy of Vitamin Therapy in lowering Homocysteine Levels among Stroke Patients with Hyperhomocysteinemia. J Integr Neurosci. 2024 Jan 10;23(1):3. doi: 10.31083/j.jin2301003.
35. Zhang SY, Xuan C, Zhang XC, Zhu J, Yue K, Zhao P, He GW, Lun LM, Tian QW. Association Between MTHFR Gene Common Variants, Serum Homocysteine, and Risk of Early-Onset Coronary Artery Disease: A Case-Control Study. Biochem Genet. 2020 Apr;58(2):245-256. doi: 10.1007/s10528-019-09937-x.
36. Hou N, Chen S, Chen F, Jiang M, Zhang J, Yang Y, Zhu B, Bai X, Hu Y, Huang H, Xu C. Association between premature ovarian failure, polymorphisms in MTHFR and MTRR genes and serum homocysteine concentration. Reprod Biomed Online. 2016 Apr;32(4):407-13. doi: 10.1016/j.rbmo.2016.01.009.
37. Moe KT, Woon FP, De Silva DA, Wong P, Koh TH, Kingwell B, Chin-Dusting J, Wong MC. Association of acute ischemic stroke with the MTHFR C677T polymorphism but not with NOS3 gene polymorphisms in a Singapore population. Eur J Neurol. 2008 Dec;15(12):1309-14. doi: 10.1111/j.1468-1331.
38. Wu H, Huang Q, Yu Z, Zhong Z. Association of ALDH2 rs671 and MTHFR rs1801133 polymorphisms with hypertension among Hakka people in Southern China. BMC Cardiovasc Disord. 2022 Mar 27;22(1):128. doi: 10.1186/s12872-022-02577-x.
39. Liang R, Zhou Y, Xie J, Lv W, Kang B, Liang Y, Chen Y, Li Y. [Association of C677T gene polymorphisms of methylenetetrahydrofolate reductase and plasma homocysteine level with hyperlipidemia]. Nan Fang Yi Ke Da Xue Xue Bao. 2014 Jul;34(8):1195-8. Chinese.
40. Real JT, Martinez-Hervas S, Garcia-Garcia AB, Chaves FJ, Civera M, Ascaso JF, Carmena R. Association of C677T polymorphism in MTHFR gene, high homocysteine and low HDL cholesterol plasma values in heterozygous familial hypercholesterolemia. J Atheroscler Thromb. 2009;16(6):815-20. doi: 10.5551/jat.2196.
41. Paradkar MU, Padate B, Shah SAV, Vora H, Ashavaid TF. Association of Genetic Variants with Hyperhomocysteinemia in Indian Patients with Thrombosis. Indian J Clin Biochem. 2020 Oct;35(4):465-473. doi: 10.1007/s12291-019-00846-9.
42. He X, Ma R, Li Y, Wang H, Yan Y, Mao Y, Liao S, Sun X, Guo S, Guo H. Association of H-Type Hypertension with miR-21, miR-29, and miR-199 in Kazahks of Xinjiang, China. Int J Hypertens. 2022 Sep 26;2022:4632087. doi: 10.1155/2022/4632087.
43. Chen L, Chen H, Wang X, Wei B, Wu Z, Chen S, Wang B, Huang H, Jin L. Association of homocysteine with IVF/ICSI outcomes stratified by MTHFR C677T polymorphisms: a prospective cohort study. Reprod Biomed Online. 2021 Jul;43(1):52-61. doi: 10.1016/j.rbmo.2021.04.009.
44. Mazokopakis EE, Papadomanolaki MG, Papadakis JA. Association of methylene tetrahydrofolate reductase (MTHFR) gene polymorphisms with serum folate, cobalanin and homocysteine concentrations in Greek adults. Scand J Clin Lab Invest. 2023 Apr;83(2):69-73. doi: 10.1080/00365513.2023.2167232.
45. Siddiqi SM, Liu L, Du Y, Song Y, Chen P, Li S, He Q, Zhou Z, Xu J, Bai J, Wang B, Qin X, Mehmood A, Xiuqing L, Cheng X, Shi HP. Association of MTHFR C677T, MTHFRA1298C, and MTRRA66G Gene Polymorphisms with Hyperhomocysteinemia and Its Modulation by the Combined Effect of Vitamin B12 and Folate in Chinese Population with Hypertension. J Nutr. 2025 Apr;155(4):1202-1209. doi: 10.1016/j.tjnut.2024.09.003.
46. Li Z, Yadav U, Mahemuti A, Tang BP, Upur H. Association of MTHFR genetic polymorphisms with venous thromboembolism in Uyghur population in Xinjiang, China. Int J Clin Exp Med. 2015 Oct 15;8(10):17703-11.
47. Cai C, Xiao R, Van Halm-Lutterodt N, Zhen J, Huang X, Xu Y, Chen S, Yuan L. Association of MTHFR, SLC19A1 Genetic Polymorphism, Serum Folate, Vitamin B_12_ and Hcy Status with Cognitive Functions in Chinese Adults. Nutrients. 2016 Oct 24;8(10):665. doi: 10.3390/nu8100665.
48. Golbahar J, Hamidi A, Aminzadeh MA, Omrani GR. Association of plasma folate, plasma total homocysteine, but not methylenetetrahydrofolate reductase C667T polymorphism, with bone mineral density in postmenopausal Iranian women: a cross-sectional study. Bone. 2004 Sep;35(3):760-5. doi: 10.1016/j.bone.2004.04.018.
49. Yingdong Zhang, Zhigang Zhu, Yang Liu. Association of plasma homocysteine level and N^5^, N^10^ – methylenetetrahydrofolate reductase gene polymorphism with cerebral infarction %J Chinese Medical Science Journal (2002). Vol.17, No.4 P.231-5.
50. Araki A, Hosoi T, Orimo H, Ito H. Association of plasma homocysteine with serum interleukin-6 and C-peptide levels in patients with type 2 diabetes. Metabolism. 2005 Jun;54(6):809-14. doi: 10.1016/j.metabol.2005.02.001.
51. Alam MA, Husain SA, Narang R, Chauhan SS, Kabra M, Vasisht S. Association of polymorphism in the thermolabile 5, 10-methylene tetrahydrofolate reductase gene and hyperhomocysteinemia with coronary artery disease. Mol Cell Biochem. 2008 Mar;310(1-2):111-7. doi: 10.1007/s11010-007-9671-7.
52. Bouzidi N, Hassine M, Fodha H, Ben Messaoud M, Maatouk F, Gamra H, Ferchichi S. Association of the methylene-tetrahydrofolate reductase gene rs1801133 C677T variant with serum homocysteine levels, and the severity of coronary artery disease. Sci Rep. 2020 Jun 22;10(1):10064. doi: 10.1038/s41598-020-66937-3.
53. Ni G, Qin J, Chen Z, Li H, Zhou J, Huang M, Zhou L. Associations between genetic variation in one-carbon metabolism and leukocyte DNA methylation in valproate-treated patients with epilepsy. Clin Nutr. 2018 Feb;37(1):308-312. doi: 10.1016/j.clnu.2017.01.004.
54. Zhu J, Wang Z, Sun X, Wang D, Xu X, Yang L, Du J, Zhou Z, Qi Y, Ma L. Associations between one-carbon metabolism and valproic acid-induced liver dysfunction in epileptic patients. Front Pharmacol. 2024 Feb 23;15:1358262. doi: 10.3389/fphar.2024.1358262.
55. Yuan X, Wang T, Gao J, Wang Y, Chen Y, Kaliannan K, Li X, Xiao J, Ma T, Zhang L, Shao Z. Associations of homocysteine status and homocysteine metabolism enzyme polymorphisms with hypertension and dyslipidemia in a Chinese hypertensive population. Clin Exp Hypertens. 2020;42(1):52-60. doi: 10.1080/10641963.2019.1571599.
56. Wang XB, Qiao C, Wei L, Han YD, Cui NH, Huang ZL, Li ZH, Zheng F, Yan M. Associations of Polymorphisms in MTHFR Gene with the Risk of Age-Related Cataract in Chinese Han Population: A Genotype-Phenotype Analysis. PLoS One. 2015 Dec 21;10(12):e0145581. doi: 10.1371/journal.pone.0145581.
57. Liu CS, Chen CH, Chiang HC, Kuo CL, Huang CS, Cheng WL, Wei YH, Chen HW. B-group vitamins, MTHFR C677T polymorphism and carotid intima-media thickness in clinically healthy subjects. Eur J Clin Nutr. 2007 Aug;61(8):996-1003. doi: 10.1038/sj.ejcn.1602606.
58. Cheng CH, Huang YC, Chen FP, Chou MC, Tsai TP. B-vitamins, homocysteine and gene polymorphism in adults with fasting or post-methionine loading hyperhomocysteinemia. Eur J Nutr. 2008 Dec;47(8):491-8. doi: 10.1007/s00394-008-0752-5.
59. Yin G, Yan L, Zhang Z, Chen K, Jin X. C677T methylenetetrahydrofolate reductase gene polymorphism as a risk factor involved in venous thromboembolism: a population-based case-control study. Mol Med Rep. 2012 Dec;6(6):1271-5. doi: 10.3892/mmr.2012.1086.
60. Kajanachumpol S, Atamasirikul K, Tantibhedhyangkul P. C677T methylenetetrahydrofolate reductase and plasma homocysteine levels among Thai vegans and omnivores. Int J Vitam Nutr Res. 2013;83(2):86-91. doi: 10.1024/0300-9831/a000148.
61. Elsaid HH, El-Hefnawy KA, Elalawi SM. *C677T MTHFR* Gene Polymorphism is Contributing Factor in Development of Renal Impairment in Young Hypertensive Patients. Indian J Clin Biochem. 2021 Apr;36(2):213-220. doi: 10.1007/s12291-020-00890-w.
62. Pethig K, Hoffmann A, Heublein B, Timke A, Gross G, Haverich A. Cardiac allograft vascular disease after orthotopic heart transplantation: methylenetetrahydrofolate reductase gene polymorphism C677T does not account for rapidly progressive forms. Transplantation. 2000 Feb 15;69(3):442-5. doi: 10.1097/00007890-200002150-00025.
63. Gorgone G, Currò M, Ferlazzo N, Parisi G, Parnetti L, Belcastro V, Tambasco N, Rossi A, Pisani F, Calabresi P, Ientile R, Caccamo D. Coenzyme Q10, hyperhomocysteinemia and MTHFR C677T polymorphism in levodopa-treated Parkinson's disease patients. Neuromolecular Med. 2012 Mar;14(1):84-90. doi: 10.1007/s12017-012-8174-1.
64. Almeida OP, Flicker L, Lautenschlager NT, Leedman P, Vasikaran S, van Bockxmeer FM. Contribution of the MTHFR gene to the causal pathway for depression, anxiety and cognitive impairment in later life. Neurobiol Aging. 2005 Feb;26(2):251-7. doi: 10.1016/j.neurobiolaging.2004.03.007.
65. Xie C, Ping P, Ma Y, Wu Z, Chen X. Correlation between methylenetetrahydrofolate reductase gene polymorphism and oligoasthenospermia and the effects of folic acid supplementation on semen quality. Transl Androl Urol. 2019 Dec;8(6):678-685. doi: 10.21037/tau.2019.11.17.
66. Zhang L, Sun L, Wei T. Correlation between MTHFR gene polymorphism and homocysteine levels for prognosis in patients with pregnancy-induced hypertension. Am J Transl Res. 2021 Jul 15;13(7):8253-8261.
67. Wu X, Liu K, Zhao X, Zhang X, Guo H, Jiang H, Chang J, Lv X, Gao X, Zhi X, Ren C, Chen Q, Liang Y, Li Y. Correlation Between the MTHFR C677T Genotype and Coronary Heart Disease in Populations from Gansu, China. DNA Cell Biol. 2022 Nov;41(11):981-986. doi: 10.1089/dna.2022.0329.
68. Li M, Fu B, Dong W. Correlations between plasma homocysteine and MTHFR gene polymorphism and white matter lesions. Folia Neuropathol. 2018;56(4):301-307. doi: 10.5114/fn.2018.80863.
69. Ji Y, Kong X, Wang G, Hong X, Xu X, Chen Z, Bartell T, Xu X, Tang G, Hou F, Huo Y, Wang X, Wang B. Distribution and determinants of plasma homocysteine levels in rural Chinese twins across the lifespan. Nutrients. 2014 Dec 18;6(12):5900-14. doi: 10.3390/nu6125900.
70. Toffoli G, Russo A, Innocenti F, Corona G, Tumolo S, Sartor F, Mini E, Boiocchi M. Effect of methylenetetrahydrofolate reductase 677C-->T polymorphism on toxicity and homocysteine plasma level after chronic methotrexate treatment of ovarian cancer patients. Int J Cancer. 2003 Jan 20;103(3):294-9. doi: 10.1002/ijc.10847.
71. Ma L, Li J, Yuan Y, Chen W, Zhao J. Effect of methylenetetrahydrofolate reductase C677T polymorphism on serum folate but not vitamin B12 levels in patients with H-type hypertension. Mol Biol Rep. 2022 Oct;49(10):9535-9541. doi: 10.1007/s11033-022-07844-w.
72. Dell'edera D, Tinelli A, Milazzo GN, Malvasi A, Domenico C, Pacella E, Pierluigi C, Giuseppe T, Marcello G, Francesco L, Epifania AA. Effect of multivitamins on plasma homocysteine in patients with the 5,10 methylenetetrahydrofolate reductase C677T homozygous state. Mol Med Rep. 2013 Aug;8(2):609-12. doi: 10.3892/mmr.2013.1563.
73. Jiang S, Chen Q, Venners SA, Zhong G, Hsu YH, Xing H, Wang X, Xu X. Effect of simvastatin on plasma homocysteine levels and its modification by MTHFR C677T polymorphism in Chinese patients with primary hyperlipidemia. Cardiovasc Ther. 2013 Aug;31(4):e27-33. doi: 10.1111/1755-5922.12002.
74. Petr M, Steffl M, Kohlíková E. Effect of the MTHFR 677C/T polymorphism on homocysteinemia in response to creatine supplementation: a case study. Physiol Res. 2013;62(6):721-9. doi: 10.33549/physiolres.932542.
75. Hekmati Azar Mehrabani Z, Ghorbanihaghjo A, Sayyah Melli M, Hamzeh-Mivehroud M, Fathi Maroufi N, Bargahi N, Bannazadeh Amirkhiz M, Rashtchizadeh N. Effects of folic acid supplementation on serum homocysteine and lipoprotein (a) levels during pregnancy. Bioimpacts. 2015;5(4):177-82. doi: 10.15171/bi.2015.26.
76. Ho V, Massey TE, King WD. Effects of methionine synthase and methylenetetrahydrofolate reductase gene polymorphisms on markers of one-carbon metabolism. Genes Nutr. 2013 Nov;8(6):571-80. doi: 10.1007/s12263-013-0358-2.
77. Jiang S, Pan M, Wu S, Venners SA, Zhong G, Hsu YH, Weinstock J, Wang B, Tang G, Liu D, Xu X. Elevation in Total Homocysteine Levels in Chinese Patients With Essential Hypertension Treated With Antihypertensive Benazepril. Clin Appl Thromb Hemost. 2016 Mar;22(2):191-8. doi: 10.1177/1076029614565881.
78. Peterlana D, Puccetti A, Caramaschi P, Biasi D, Beri R, Simeoni S, Corrocher R, Lunardi C. Endothelin-1 serum levels correlate with MCP-1 but not with homocysteine plasma concentration in patients with systemic sclerosis. Scand J Rheumatol. 2006 Mar-Apr;35(2):133-7. doi: 10.1080/03009740500385584.
79. Vurucu S, Demirkaya E, Kul M, Unay B, Gul D, Akin R, Gokçay E. Evaluation of the relationship between C677T variants of methylenetetrahydrofolate reductase gene and hyperhomocysteinemia in children receiving antiepileptic drug therapy. Prog Neuropsychopharmacol Biol Psychiatry. 2008 Apr 1;32(3):844-8. doi: 10.1016/j.pnpbp.2007.12.018.
80. Liang X, He T, Gao L, Wei L, Rong D, Zhang Y, Liu Y. Explore the Role of the rs1801133-PPARG Pathway in the H-type Hypertension. PPAR Res. 2022 Mar 20;2022:2054876. doi: 10.1155/2022/2054876.
81. You X, Zhang Z, Xu Y, Yang B, Huang S, Zou Y, Zhao F, Feng C, Lao H, Yuan H, Liu Y, Wu M. Exploring the correlation between homocysteine, red blood cell folate and MTHFRC677T genotypes with female infertility. Biomark Med. 2024;18(17-18):749-758. doi: 10.1080/17520363.2024.2394386.
82. Candito M, Bedoucha P, Gibelin P, Jambou D, de Franchis R, Sadoul JL, Chatel M, Van Obberghen E. Fasting, postprandial, and post-methionine-load homocysteinaemia and methylenetetrahydrofolate reductase polymorphism in vascular disease. J Inherit Metab Dis. 1999 Jun;22(5):588-92. doi: 10.1023/a:1005513626542.
83. Li WX, Cheng F, Zhang AJ, Dai SX, Li GH, Lv WW, Zhou T, Zhang Q, Zhang H, Zhang T, Liu F, Liu D, Huang JF. Folate Deficiency and Gene Polymorphisms of MTHFR, MTR and MTRR Elevate the Hyperhomocysteinemia Risk. Clin Lab. 2017 Mar 1;63(3):523-533. doi: 10.7754/Clin.Lab.2016.160917.
84. Barnabé A, Aléssio AC, Bittar LF, de Moraes Mazetto B, Bicudo AM, de Paula EV, Höehr NF, Annichino-Bizzacchi JM. Folate, vitamin B12 and Homocysteine status in the post-folic acid fortification era in different subgroups of the Brazilian population attended to at a public health care center. Nutr J. 2015 Feb 19;14:19. doi: 10.1186/s12937-015-0006-3.
85. Benke K, Ágg B, Mátyás G, Szokolai V, Harsányi G, Szilveszter B, Odler B, Pólos M, Maurovich-Horvat P, Radovits T, Merkely B, Nagy ZB, Szabolcs Z. Gene polymorphisms as risk factors for predicting the cardiovascular manifestations in Marfan syndrome. Role of folic acid metabolism enzyme gene polymorphisms in Marfan syndrome. Thromb Haemost. 2015 Oct;114(4):748-56. doi: 10.1160/TH15-02-0096.
86. Botto N, Andreassi MG, Manfredi S, Masetti S, Cocci F, Colombo MG, Storti S, Rizza A, Biagini A. Genetic polymorphisms in folate and homocysteine metabolism as risk factors for DNA damage. Eur J Hum Genet. 2003 Sep;11(9):671-8. doi: 10.1038/sj.ejhg.5201024.
87. Nursal AF, Yigit S, Rustemoglu H, Cenikli A. A Case-Control Study Investigating the Effect of MTHFR C677T Variant on Performance of Elite Athletes. Endocr Metab Immune Disord Drug Targets. 2021;21(9):1685-1690. doi: 10.2174/1568026620666201022144819.
88. Zhang Y, Zhang H, Wang J, Wei X, Qu YI, Xu F, Zhang L. A genetic variant study of bortezomib-induced peripheral neuropathy in Chinese multiple myeloma patients. Oncol Res. 2024 Apr 23;32(5):955-963. doi: 10.32604/or.2023.043922.
89. Kostulas K, Crisby M, Huang WX, Lannfelt L, Hagenfeldt L, Eggertsen G, Kostulas V, Hillert J. A methylenetetrahydrofolate reductase gene polymorphism in ischaemic stroke and in carotid artery stenosis. Eur J Clin Invest. 1998 Apr;28(4):285-9. doi: 10.1046/j.1365-2362.1998.00281.x.
90. Ye F, Zhang S, Qi Q, Zhou J, Du Y, Wang L. Association of MTHFR 677C>T polymorphism with pregnancy outcomes in IVF/ICSI-ET recipients with adequate synthetic folic acid supplementation. Biosci Trends. 2022 Sep 17;16(4):282-290. doi: 10.5582/bst.2021.01306.
91. Lucock M, Daskalakis I, Briggs D, Yates Z, Levene M. Altered folate metabolism and disposition in mothers affected by a spina bifida pregnancy: influence of 677c --> t methylenetetrahydrofolate reductase and 2756a --> g methionine synthase genotypes. Mol Genet Metab. 2000 May;70(1):27-44. doi: 10.1006/mgme.2000.2994.
92. Kawamoto R, Kohara K, Tabara Y, Miki T, Doi T, Tokunaga H, Konishi I. An association of 5,10-methylenetetrahydrofolate reductase (MTHFR) gene polymorphism and common carotid atherosclerosis. J Hum Genet. 2001;46(9):506-10. doi: 10.1007/s100380170031.
93. Kawamoto R, Kohara K, Oka Y, Tomita H, Tabara Y, Miki T. An association of 5,10-methylenetetrahydrofolate reductase (MTHFR) gene polymorphism and ischemic stroke. J Stroke Cerebrovasc Dis. 2005 Mar-Apr;14(2):67-74. doi: 10.1016/j.jstrokecerebrovasdis.
94. Saffari B, Senemar S, Karimi M, Bahari M, Jooyan N, Yavarian M. An MTHFR variant, plasma homocysteine levels and late-onset coronary artery disease in subjects from southern Iran. Pak J Biol Sci. 2013 Aug 15;16(16):788-95. doi: 10.3923/pjbs.2013.788.795.
95. Tang Z, Xiao L, Wang JQ, Zhang T. Analysis of metabolism-related indicators and MTHFR gene polymorphism in patients with H-type hypertension. Minerva Med. 2017 Apr;108(2):103-107. doi: 10.23736/S0026-4806.16.04951-X.
96. Terruzzi I, Senesi P, Fermo I, Lattuada G, Luzi L. Are genetic variants of the methyl group metabolism enzymes risk factors predisposing to obesity? J Endocrinol Invest. 2007 Oct;30(9):747-53. doi: 10.1007/BF03350812.
97. Chorąży M, Wawrusiewicz-Kurylonek N, Gościk J, Posmyk R, Czarnowska A, Więsik M, Kapica-Topczewska K, Krętowski AJ, Kochanowicz J, Kułakowska A. Association between polymorphisms of a folate - homocysteine - methionine - SAM metabolising enzyme gene and multiple sclerosis in a Polish population. Neurol Neurochir Pol. 2019;53(3):194-198. doi: 10.5603/PJNNS.a2019.0019.
98. Liang Z, Fan F, Liu B, Li K, Chen H, Jia J, Huo Y, Li J, Zhang Y. Association Between Serum Folate Concentrations and 10-Year Stroke Risk in a Prospective Community Cohort: Mediation and Interaction Analyses. Nutrients. 2024 Dec 31;17(1):159. doi: 10.3390/nu17010159.
99. Wang Y, Zhang J, Qian Y, Tang X, Ling H, Chen K, Li Y, Gao P, Zhu D. Association of Homocysteine with Aysmptomatic Intracranial and Extracranial Arterial Stenosis in Hypertension Patients. Sci Rep. 2018 Jan 12;8(1):595. doi: 10.1038/s41598-017-19125-9.
100. Chaabane S, Messedi M, Akrout R, Ben Hamad M, Turki M, Marzouk S, Keskes L, Bahloul Z, Rebai A, Ayedi F, Maalej A. Association of hyperhomocysteinemia with genetic variants in key enzymes of homocysteine metabolism and methotrexate toxicity in rheumatoid arthritis patients. Inflamm Res. 2018 Aug;67(8):703-710. doi: 10.1007/s00011-018-1161-8.
101. Pandey SK, Singh A, Polipalli SK, Gupta S, Kapoor S. Association of Methylene Tetrahydrofolate Reductase Polymorphism with BMD and Homocysteine in Premenopausal North Indian Women. J Clin Diagn Res. 2013 Dec;7(12):2908-11. doi: 10.7860/JCDR/2013/6670.3722.
102. Luo J, Chen X, Yang Y, Liu Y, Feng Y, Chen G. Association of MTHFR C667T Polymorphism, Homocysteine, and B Vitamins with Senile Cataract. J Nutr Sci Vitaminol (Tokyo). 2023;69(2):136-144. doi: 10.3177/jnsv.69.136.
103. Wang B, Jin F, Kan R, Ji S, Zhang C, Lu Z, Zheng C, Yang Z, Wang L. Association of MTHFR gene polymorphism C677T with susceptibility to late-onset Alzheimer's disease. J Mol Neurosci. 2005;27(1):23-7. doi: 10.1385/JMN:27:1:023.
104. Buendía-Pazarán JG, Hernández-Zamora E, Rodríguez-Olivas AO, Casas-Ávila L, Valdés-Flores M, Reyes-Maldonado E. Association of MTHFR rs1801133 and homocysteine with Legg-Calvé-Perthes disease in Mexican patients. Orphanet J Rare Dis. 2022 Mar 9;17(1):123. doi: 10.1186/s13023-022-02264-2.
105. Cai CQ, Fang YL, Shu JB, Zhao LS, Zhang RP, Cao LR, Wang YZ, Zhi XF, Cui HL, Shi OY, Liu W. Association of neural tube defects with maternal alterations and genetic polymorphisms in one-carbon metabolic pathway. Ital J Pediatr. 2019 Mar 14;45(1):37. doi: 10.1186/s13052-019-0630-1.
106. Liu S, Liu M, Li Q, Liu X, Wang Y, Mambiya M, Zhang K, Yang L, Zhang Q, Shang M, Zeng F, Nie F, Liu W. Association of single nucleotide polymorphisms of MTHFR, TCN2, RNF213 with susceptibility to hypertension and blood pressure. Biosci Rep. 2019 Dec 20;39(12):BSR20191454. doi: 10.1042/BSR20191454.
107. Al-Absi IK, Al-Subaie AM, Ameen G, Mahdi N, Mohammad AM, Fawaz NA, Almawi WY. Association of the methylenetetrahydrofolate reductase A1298C but not the C677T single nucleotide polymorphism with sickle cell disease in Bahrain. Hemoglobin. 2006;30(4):449-53. doi: 10.1080/03630260600867958.
108. Chen L, Jiang Y. Association Study of *MTHFR* C677T Polymorphism With Homocysteine Level and Coronary Heart Disease in Elderly Patients. Cardiol Res Pract. 2025 Mar 16;2025:6246458. doi: 10.1155/crp/6246458.
109. Yang BY, Cao K, Luo YN, He ZZ, Guo PY, Ma HM, Yang M, Zhou Y, Hu LW, Chen GB, Zeng XW, Yu HY, Yu Y, Dong GH. Associations of ambient particulate matter with homocysteine metabolism markers and effect modification by B vitamins and MTHFR C677T gene polymorphism. Environ Pollut. 2021 Feb 1;270:116211. doi: 10.1016/j.envpol.2020.116211.
110. Krumdieck CL, Fernandez JR, Desmond RA, Kleinstein RN, Shipp MD, Prince CW. C677T methylene-H4-folate-reductase variant decreases binocular accommodation. Optom Vis Sci. 2008 Mar;85(3):196-200. doi: 10.1097/OPX.0b013e3181643e97.
111. Boraldi F, Costa S, Rabacchi C, Ciani M, Vanakker O, Quaglino D. Can APOE and MTHFR polymorphisms have an influence on the severity of cardiovascular manifestations in Italian Pseudoxanthoma elasticum affected patients? Mol Genet Metab Rep. 2014 Nov 16;1:477-482. doi: 10.1016/j.ymgmr.2014.11.002.
112. Huang LQ, Wu CX, Wei HQ, Xu G. Clinical characteristics of H-type hypertension and its relationship with the MTHFR C677T polymorphism in a Zhuang population from Guangxi, China. J Clin Lab Anal. 2020 Nov;34(11):e23499. doi: 10.1002/jcla.23499.
113. Luo M, Ji H, Zhou X, Liang J, Zou T. Correlation of homocysteine metabolic enzymes gene polymorphism and mild cognitive impairment in the Xinjiang Uygur population. Med Sci Monit. 2015 Jan 27;21:326-32. doi: 10.12659/MSM.893226.
114. Torres-Sánchez L, Chen J, Díaz-Sánchez Y, Palomeque C, Bottiglieri T, López-Cervantes M, López-Carrillo L. Dietary and genetic determinants of homocysteine levels among Mexican women of reproductive age. Eur J Clin Nutr. 2006 Jun;60(6):691-7. doi: 10.1038/sj.ejcn.1602370.
115. Guo QN, Wang L, Liu ZY, Wang HD, Wang L, Long JG, Liao SX. Different effects of maternal homocysteine concentration, MTHFR and MTRR genetic polymorphisms on the occurrence of fetal aneuploidy. Reprod Biomed Online. 2022 Dec;45(6):1207-1215. doi: 10.1016/j.rbmo.2022.06.024.
116. Bahadır A, Eroz R, Türker Y. Does the MTHFR C677T gene polymorphism indicate cardiovascular disease risk in type 2 diabetes mellitus patients? Anatol J Cardiol. 2015 Jul;15(7):524-30. doi: 10.5152/akd.2014.5555.
117. Pereira AC, Miyakawa AA, Lopes NH, Soares PR, de Oliveira SA, Cesar LA, Ramires JF, Hueb W, Krieger JE. Dynamic regulation of MTHFR mRNA expression and C677T genotype modulate mortality in coronary artery disease patients after revascularization. Thromb Res. 2007;121(1):25-32. doi: 10.1016/j.thromres.2007.03.004.
118. Zhang S, Wang T, Wang H, Tang J, Hou A, Yan X, Yu B, Ran S, Luo M, Tang Y, Yang R, Song D, He H. Effects of individualized administration of folic acid on prothrombotic state and vascular endothelial function with H-type hypertension: A double-blinded, randomized clinical cohort study. Medicine (Baltimore). 2022 Jan 21;101(3):e28628. doi: 10.1097/MD.0000000000028628.
119. Chan CYT, Cheng SWK. Elevated homocysteine in human abdominal aortic aneurysmal tissues. Vasc Med. 2017 Oct;22(5):370-377. doi: 10.1177/1358863X17718260.
120. Fatini C, Sofi F, Gori AM, Sticchi E, Marcucci R, Lenti M, Casini A, Surrenti C, Abbate R, Gensini GF. Endothelial nitric oxide synthase -786T>C, but not 894G>T and 4a4b, polymorphism influences plasma homocysteine concentrations in persons with normal vitamin status. Clin Chem. 2005 Jul;51(7):1159-64. doi: 10.1373/clinchem.2005.048850.
121. Chen NY, Liu CW, Du LL, Xiao LP, Ge L, Wang YY, Wei Z, Wu HY, Luo CY, Liang L, Peng JH, Luo XQ, Yin RX, Nguyen CP, Pan SL. Enrichment of MTHFR 677 T in a Chinese long-lived cohort and its association with lipid modulation. Lipids Health Dis. 2014 Jun 26;13:104. doi: 10.1186/1476-511X-13-104.
122. Li WX, Li W, Cao JQ, Yan H, Sun Y, Zhang H, Zhang Q, Tang L, Wang M, Huang JF, Liu D. Folate Deficiency Was Associated with Increased Alanine Aminotransferase and Glutamyl Transpeptidase Concentrations in a Chinese Hypertensive Population: A Cross-Sectional Study. J Nutr Sci Vitaminol (Tokyo). 2016;62(4):265-271. doi: 10.3177/jnsv.62.265.
123. Guedes T, Santos AA, Vieira-Neto FH, Bianco B, Barbosa CP, Christofolini DM. Folate metabolism abnormalities in infertile patients with endometriosis. Biomark Med. 2022 May;16(7):549-557. doi: 10.2217/bmm-2021-0076.
124. Ng X, Boyd L, Dufficy L, Naumovski N, Blades B, Travers C, Lewis P, Sturm J, Yates Z, Townley-Jones M, Roach P, Veysey M, Lucock M. Folate nutritional genetics and risk for hypertension in an elderly population sample. J Nutrigenet Nutrigenomics. 2009;2(1):1-8. doi: 10.1159/000160079.
125. Martín I, Obrador A, Gibert MJ, Hernanz A, Fuster A, Pintos C, Garcia A, Tur J. Folate status and a new repletion cut-off value in a group of healthy Majorcan women. Clin Nutr. 2003 Feb;22(1):53-8. doi: 10.1054/clnu.2002.0591.
126. Bostom AG, Shemin D, Lapane KL, Nadeau MR, Sutherland P, Chan J, Rozen R, Yoburn D, Jacques PF, Selhub J, Rosenberg IH. Folate status is the major determinant of fasting total plasma homocysteine levels in maintenance dialysis patients. Atherosclerosis. 1996 Jun;123(1-2):193-202. doi: 10.1016/0021-9150(96)05809-1.
127. Nienaber-Rousseau C, Ellis SM, Moss SJ, Melse-Boonstra A, Towers GW. Gene-environment and gene-gene interactions of specific MTHFR, MTR and CBS gene variants in relation to homocysteine in black South Africans. Gene. 2013 Nov 1;530(1):113-8. doi: 10.1016/j.gene.2013.07.065.
128. Zhang G, Dai C. Gene polymorphisms of homocysteine metabolism-related enzymes in Chinese patients with occlusive coronary artery or cerebral vascular diseases. Thromb Res. 2001 Nov 1;104(3):187-95. doi: 10.1016/s0049-3848(01)00352-8.
129. Summers CM, Mitchell LE, Stanislawska-Sachadyn A, Baido SF, Blair IA, Von Feldt JM, Whitehead AS. Genetic and lifestyle variables associated with homocysteine concentrations and the distribution of folate derivatives in healthy premenopausal women. Birth Defects Res A Clin Mol Teratol. 2010 Aug;88(8):679-88. doi: 10.1002/bdra.20683.
130. Marcucci R, Giusti B, Betti I, Evangelisti L, Fedi S, Sodi A, Cappelli S, Menchini U, Abbate R, Prisco D. Genetic determinants of fasting and post-methionine hyperhomocysteinemia in patients with retinal vein occlusion. Thromb Res. 2003 Apr 15;110(1):7-12. doi: 10.1016/s0049-3848(03)00293-7.
131. Chen JX, Shi Q, Wang XW, Guo S, Dai W, Li K, Song P, Wei C, Wang G, Li CY, Gao TW. Genetic polymorphisms in the methylenetetrahydrofolate reductase gene (MTHFR) and risk of vitiligo in Han Chinese populations: a genotype-phenotype correlation study. Br J Dermatol. 2014 May;170(5):1092-9. doi: 10.1111/bjd.12845.
132. Biselli JM, Goloni-Bertollo EM, Zampieri BL, Haddad R, Eberlin MN, Pavarino-Bertelli EC. Genetic polymorphisms involved in folate metabolism and elevated plasma concentrations of homocysteine: maternal risk factors for Down syndrome in Brazil. Genet Mol Res. 2008 Jan 22;7(1):33-42. doi: 10.4238/vol7-1gmr388.
133. Biselli JM, Zampieri BL, Goloni-Bertollo EM, Haddad R, Fonseca MF, Eberlin MN, Vannucchi H, Carvalho VM, Pavarino EC. Genetic polymorphisms modulate the folate metabolism of Brazilian individuals with Down syndrome. Mol Biol Rep. 2012 Oct;39(10):9277-84. doi: 10.1007/s11033-012-1629-5.
134. Du B, Tian H, Tian D, Zhang C, Wang W, Wang L, Ge M, Hou Q, Zhang W. Genetic polymorphisms of key enzymes in folate metabolism affect the efficacy of folate therapy in patients with hyperhomocysteinaemia. Br J Nutr. 2018 Apr;119(8):887-895. doi: 10.1017/S0007114518000508.
135. Miriuka SG, Langman LJ, Evrovski J, Miner SE, D'Mello N, Delgado DH, Wong BY, Ross HJ, Cole DE. Genetic polymorphisms predisposing to hyperhomocysteinemia in cardiac transplant patients. Transpl Int. 2005 Jan;18(1):29-35. doi: 10.1111/j.1432-2277.2004.00021.x.
136. Gromadzka G, Rudnicka M, Chabik G, Przybyłkowski A, Członkowska A. Genetic variability in the methylenetetrahydrofolate reductase gene (MTHFR) affects clinical expression of Wilson's disease. J Hepatol. 2011 Oct;55(4):913-9. doi: 10.1016/j.jhep.2011.01.030.
137. Aşcı AE, Orhan G, Karahalil B. Genetic variants of folate metabolism and the risk of multiple sclerosis. Neurol Res. 2024 Jun;46(6):544-552. doi: 10.1080/01616412.2024.2337519.
138. Yang B, Liu Y, Li Y, Fan S, Zhi X, Lu X, Wang D, Zheng Q, Wang Y, Wang Y, Sun G. Geographical distribution of MTHFR C677T, A1298C and MTRR A66G gene polymorphisms in China: findings from 15357 adults of Han nationality. PLoS One. 2013;8(3):e57917. doi: 10.1371/journal.pone.0057917.
139. Zheng W, Zhang Y, Zhang P, Chen T, Yan X, Li L, Shao L, Song Z, Han W, Wang J, Huang J, Ma K, Yang R, Ma Y, Xu L, Zhang K, Yuan X, Li G. Gestational diabetes mellitus is associated with distinct folate-related metabolites in early and mid-pregnancy: A prospective cohort study. Diabetes Metab Res Rev. 2024 May;40(4):e3814. doi: 10.1002/dmrr.3814.
140. du Plessis JP, Lammertyn L, Schutte AE, Nienaber-Rousseau C. H-Type Hypertension among Black South Africans and the Relationship between Homocysteine, Its Genetic Determinants and Estimates of Vascular Function. J Cardiovasc Dev Dis. 2022 Dec 9;9(12):447. doi: 10.3390/jcdd9120447.
141. Sofi F, Marcucci R, Giusti B, Pratesi G, Lari B, Sestini I, Lo Sapio P, Pulli R, Pratesi C, Abbate R, Gensini GF. High levels of homocysteine, lipoprotein (a) and plasminogen activator inhibitor-1 are present in patients with abdominal aortic aneurysm. Thromb Haemost. 2005 Nov;94(5):1094-8.
142. Flicker L, Martins RN, Thomas J, Acres J, Taddei K, Norman P, Jamrozik K, Almeida OP. Homocysteine, Alzheimer genes and proteins, and measures of cognition and depression in older men. J Alzheimers Dis. 2004 Jun;6(3):329-36. doi: 10.3233/jad-2004-6313.
143. Rodriguez-Oroz MC, Lage PM, Sanchez-Mut J, Lamet I, Pagonabarraga J, Toledo JB, García-Garcia D, Clavero P, Samaranch L, Irurzun C, Matsubara JM, Irigoien J, Bescos E, Kulisevsky J, Pérez-Tur J, Obeso JA. Homocysteine and cognitive impairment in Parkinson's disease: a biochemical, neuroimaging, and genetic study. Mov Disord. 2009 Jul 30;24(10):1437-44. doi: 10.1002/mds.22522.
144. Guerzoni AR, Biselli PM, Godoy MF, Souza DR, Haddad R, Eberlin MN, Pavarino-Bertelli EC, Goloni-Bertollo EM. Homocysteine and MTHFR and VEGF gene polymorphisms: impact on coronary artery disease. Arq Bras Cardiol. 2009 Apr;92(4):263-8. English, Portuguese, Spanish. doi: 10.1590/s0066-782x2009000400003.
145. Nagaraja D, Noone ML, Bharatkumar VP, Christopher R. Homocysteine, folate and vitamin B(12) in puerperal cerebral venous thrombosis. J Neurol Sci. 2008 Sep 15;272(1-2):43-7. doi: 10.1016/j.jns.2008.03.021.
146. Camicioli RM, Bouchard TP, Somerville MJ. Homocysteine is not associated with global motor or cognitive measures in nondemented older Parkinson's disease patients. Mov Disord. 2009 Jan 30;24(2):176-82. doi: 10.1002/mds.22227.
147. Orio F Jr, Palomba S, Di Biase S, Colao A, Tauchmanova L, Savastano S, Labella D, Russo T, Zullo F, Lombardi G. Homocysteine levels and C677T polymorphism of methylenetetrahydrofolate reductase in women with polycystic ovary syndrome. J Clin Endocrinol Metab. 2003 Feb;88(2):673-9.
148. Zappacosta B, Mastroiacovo P, Persichilli S, Pounis G, Ruggeri S, Minucci A, Carnovale E, Andria G, Ricci R, Scala I, Genovese O, Turrini A, Mistura L, Giardina B, Iacoviello L. Homocysteine lowering by folate-rich diet or pharmacological supplementations in subjects with moderate hyperhomocysteinemia. Nutrients. 2013 May 8;5(5):1531-43. doi: 10.3390/nu5051531.
149. Caldeira-Araújo H, Ramos R, Florindo C, Rivera I, Castro R, Tavares de Almeida I. Homocysteine Metabolism in Children and Adolescents: Influence of Age on Plasma Biomarkers and Correspondent Genotype Interactions. Nutrients. 2019 Mar 16;11(3):646. doi: 10.3390/nu11030646.
150. Herrmann W, Obeid R, Schorr H, Zarzour W, Geisel J. Homocysteine, methylenetetrahydrofolate reductase C677T polymorphism and the B-vitamins: a facet of nature-nurture interplay. Clin Chem Lab Med. 2003 Apr;41(4):547-53. doi: 10.1515/CCLM.2003.083.
151. Bjerregaard LT, Nederby NJ, Fredholm L, Brandslund I, Munkholm P, Hey H. Hyperhomocysteinaemia, coagulation pathway activation and thrombophilia in patients with inflammatory bowel disease. Scand J Gastroenterol. 2002 Jan;37(1):62-7. doi: 10.1080/003655202753387374.
152. He JA, Hu XH, Fan YY, Yang J, Zhang ZS, Liu CW, Yang DH, Zhang J, Xin SJ, Zhang Q, Duan ZQ. Hyperhomocysteinaemia, low folate concentrations and methylene tetrahydrofolate reductase C677T mutation in acute mesenteric venous thrombosis. Eur J Vasc Endovasc Surg. 2010 Apr;39(4):508-13. doi: 10.1016/j.ejvs.2009.09.014.
153. Cao H, Hu X, Zhang Q, Li J, Liu B, Wang J, Shao Y, Zhang Z, Liu C, Hu H, Zhang J, Xin S. Hyperhomocysteinaemia, low folate concentrations and MTHFR C677T mutation in abdominal aortic aneurysm. Vasa. 2014 May;43(3):181-8. doi: 10.1024/0301-1526/a000347.
154. Sottilotta G, Siboni SM, Latella C, Oriana V, Romeo E, Santoro R, Consonni D, Trapani Lombardo V. Hyperhomocysteinemia and C677T MTHFR genotype in patients with retinal vein thrombosis. Clin Appl Thromb Hemost. 2010 Oct;16(5):549-53. doi: 10.1177/1076029609348644.
155. Biagini MR, Tozzi A, Marcucci R, Paniccia R, Fedi S, Milani S, Galli A, Ceni E, Capanni M, Manta R, Abbate R, Surrenti C. Hyperhomocysteinemia and hypercoagulability in primary biliary cirrhosis. World J Gastroenterol. 2006 Mar 14;12(10):1607-12. doi: 10.3748/wjg.v12.i10.1607.
156. Bharatkumar VP, Nagaraja D, Christopher R. Hyperhomocysteinemia and methylenetetrahydrofolate reductase C677T polymorphism in cerebral veno-sinus thrombosis. Clin Appl Thromb Hemost. 2014 Jan;20(1):78-83. doi: 10.1177/1076029612466285.
157. Ventura P, Venturelli G, Marcacci M, Fiorini M, Marchini S, Cuoghi C, Pietrangelo A. Hyperhomocysteinemia and MTHFR C677T polymorphism in patients with portal vein thrombosis complicating liver cirrhosis. Thromb Res. 2016 May;141:189-95. doi: 10.1016/j.thromres.2016.03.024.
158. Papa A, De Stefano V, Danese S, Chiusolo P, Persichilli S, Casorelli I, Zappacosta B, Giardina B, Gasbarrini A, Leone G, Gasbarrini G. Hyperhomocysteinemia and prevalence of polymorphisms of homocysteine metabolism-related enzymes in patients with inflammatory bowel disease. Am J Gastroenterol. 2001 Sep;96(9):2677-82. doi: 10.1111/j.1572-0241.2001.04127.x.
159. Herrmann W, Obeid R, Jouma M. Hyperhomocysteinemia and vitamin B-12 deficiency are more striking in Syrians than in Germans--causes and implications. Atherosclerosis. 2003 Jan;166(1):143-50. doi: 10.1016/s0021-9150(02)00320-9.
160. Salem-Berrabah OB, Mrissa R, Machghoul S, Hamida AB, N'siri B, Mazigh C, Aouni Z, Louati I, Layouni S, El Oudi M, Fekih-Mrissa N, Gritli N. Hyperhomocysteinemia, C677T MTHFR polymorphism and ischemic stroke in Tunisian patients. Tunis Med. 2010 Sep;88(9):655-9.
161. Belcastro V, Striano P, Gorgone G, Costa C, Ciampa C, Caccamo D, Pisani LR, Oteri G, Marciani MG, Aguglia U, Striano S, Ientile R, Calabresi P, Pisani F. Hyperhomocysteinemia in epileptic patients on new antiepileptic drugs. Epilepsia. 2010 Feb;51(2):274-9. doi: 10.1111/j.1528-1167.2009.02303.x.
162. Rossokha Z, Fishchuk L, Vorobei L, Medvedieva N, Popova O, Vershyhora V, Sheyko L, Brisevac L, Stroy D, Gorovenko N. Hyperhomocysteinemia in men and women of married couples with reproductive disorders. What is the difference? Syst Biol Reprod Med. 2023 Feb;69(1):75-85. doi: 10.1080/19396368.2022.2124896.
163. Gorgone G, Caccamo D, Pisani LR, Currò M, Parisi G, Oteri G, Ientile R, Rossini PM, Pisani F. Hyperhomocysteinemia in patients with epilepsy: does it play a role in the pathogenesis of brain atrophy? A preliminary report. Epilepsia. 2009 Jan;50 Suppl 1:33-6. doi: 10.1111/j.1528-1167.2008.01967.x.
164. Zarur EB, Peron Filho F, de Oliveira AC, Keppeke GD, D'Almeida V, Silva de Souza AW. Hyperhomocysteinemia in Takayasu arteritis-genetically defined or burden of the proinflammatory state? Front Immunol. 2025 Apr 4;16:1574479. doi: 10.3389/fimmu.2025.1574479.
165. Gorgone G, Ursini F, Altamura C, Bressi F, Tombini M, Curcio G, Chiovenda P, Squitti R, Silvestrini M, Ientile R, Pisani F, Rossini PM, Vernieri F. Hyperhomocysteinemia, intima-media thickness and C677T MTHFR gene polymorphism: a correlation study in patients with cognitive impairment. Atherosclerosis. 2009 Sep;206(1):309-13. doi: 10.1016/j.atherosclerosis.2009.02.028.
166. Faria-Neto JR, Chagas AC, Bydlowski SP, Lemos Neto PA, Chamone DA, Ramirez JA, da Luz PL. Hyperhomocystinemia in patients with coronary artery disease. Braz J Med Biol Res. 2006 Apr;39(4):455-63. doi: 10.1590/s0100-879x2006000400005.
167. Abd-Elmawla MA, Rizk SM, Youssry I, Shaheen AA. Impact of Genetic Polymorphism of methylenetetrahydrofolate reductase C677T on Development of Hyperhomocysteinemia and Related Oxidative Changes in Egyptian β-Thalassemia Major Patients. PLoS One. 2016 May 17;11(5):e0155070. doi: 10.1371/journal.pone.0155070.
168. Waśkiewicz A, Piotrowski W, Broda G, Sobczyk-Kopcioł A, Płoski R. Impact of MTHFR C677T gene polymorphism and vitamins intake on homocysteine concentration in the Polish adult population. Kardiol Pol. 2011;69(12):1259-64.
169. Hsu TS, Hsu LA, Chang CJ, Sun CF, Ko YL, Kuo CT, Chiang CW, Lee YS. Importance of hyperhomocysteinemia as a risk factor for venous thromboembolism in a Taiwanese population. A case-control study. Thromb Res. 2001 Jun 1;102(5):387-95. doi: 10.1016/s0049-3848(01)00262-6.
170. Strauss E, Waliszewski K, Gabriel M, Zapalski S, Pawlak AL. Increased risk of the abdominal aortic aneurysm in carriers of the MTHFR 677T allele. J Appl Genet. 2003;44(1):85-93.
171. Abd El-Aziz TA, Mohamed RH. Influence of MTHFR C677T gene polymorphism in the development of cardiovascular disease in Egyptian patients with rheumatoid arthritis. Gene. 2017 Apr 30;610:127-132. doi: 10.1016/j.gene.2017.02.015.
172. Zeng Q, Li F, Xiang T, Wang W, Ma C, Yang C, Chen H, Xiang H. Influence of food groups on plasma total homocysteine for specific MTHFR C677T genotypes in Chinese population. Mol Nutr Food Res. 2017 Feb;61(2):1600351. doi: 10.1002/mnfr.201600351.
173. El Maataoui A, Idouz K, El Maghraoui A, Nadifi S, Ouzzif Z. Influence of homocysteine and its major genetic and nutritional determinants on bone mineral density. Tunis Med. 2022 juillet;100(7):514-519.
174. Yates Z, Lucock M. Interaction between common folate polymorphisms and B-vitamin nutritional status modulates homocysteine and risk for a thrombotic event. Mol Genet Metab. 2003 Jul;79(3):201-13. doi: 10.1016/s1096-7192(03)00093-3.
175. Balogh E, Bereczky Z, Katona E, Koszegi Z, Edes I, Muszbek L, Czuriga I. Interaction between homocysteine and lipoprotein(a) increases the prevalence of coronary artery disease/myocardial infarction in women: a case-control study. Thromb Res. 2012 Feb;129(2):133-8. doi: 10.1016/j.thromres.2011.07.001.
176. Pezzini A, Grassi M, Del Zotto E, Assanelli D, Archetti S, Negrini R, Caimi L, Padovani A. Interaction of homocysteine and conventional predisposing factors on risk of ischaemic stroke in young people: consistency in phenotype-disease analysis and genotype-disease analysis. J Neurol Neurosurg Psychiatry. 2006 Oct;77(10):1150-6. doi: 10.1136/jnnp.2005.076083.
177. Amaral FM, Miranda-Vilela AL, Lordelo GS, Ribeiro IF, Daldegan MB, Grisolia CK. Interactions among methylenetetrahydrofolate reductase (MTHFR) and cystathionine β-synthase (CBS) polymorphisms - a cross-sectional study: multiple heterozygosis as a risk factor for higher homocysteine levels and vaso-occlusive episodes. Genet Mol Res. 2017 Feb 23;16(1). doi: 10.4238/gmr16019374.
178. Oliveira IO, Silva LP, Borges MC, Cruz OM, Tessmann JW, Motta JV, Seixas FK, Horta BL, Gigante DP. Interactions between lifestyle and MTHFR polymorphisms on homocysteine concentrations in young adults belonging to the 1982 Pelotas Birth Cohort. Eur J Clin Nutr. 2017 Feb;71(2):259-266. doi: 10.1038/ejcn.2016.193.
179. Fowdar JY, Lason MV, Szvetko AL, Lea RA, Griffiths LR. Investigation of homocysteine-pathway-related variants in essential hypertension. Int J Hypertens. 2012;2012:190923. doi: 10.1155/2012/190923.
180. Strauss E, Supinski W, Radziemski A, Oszkinis G, Pawlak AL, Gluszek J. Is hyperhomocysteinemia a causal factor for heart failure? The impact of the functional variants of MTHFR and PON1 on ischemic and non-ischemic etiology. Int J Cardiol. 2017 Feb 1;228:37-44. doi: 10.1016/j.ijcard.2016.11.213.
181. Mazza A, Motti C, Nulli A, Marra G, Gnasso A, Pastore A, Federici G, Cortese C. Lack of association between carotid intima-media thickness and methylenetetrahydrofolate reductase gene polymorphism or serum homocysteine in non-insulin-dependent diabetes mellitus. Metabolism. 2000 Jun;49(6):718-23. doi: 10.1053/meta.2000.6254.
182. Yazdanpanah N, Uitterlinden AG, Zillikens MC, Jhamai M, Rivadeneira F, Hofman A, de Jonge R, Lindemans J, Pols HA, van Meurs JB. Low dietary riboflavin but not folate predicts increased fracture risk in postmenopausal women homozygous for the MTHFR 677 T allele. J Bone Miner Res. 2008 Jan;23(1):86-94. doi: 10.1359/jbmr.070812.
183. Kumari P, Ali A, Sukla KK, Singh SK, Raman R. Lower incidence of nonsyndromic cleft lip with or without cleft palate in females: is homocysteine a factor? J Biosci. 2013 Mar;38(1):21-6. doi: 10.1007/s12038-013-9298-7.
184. González Ordóñez AJ, Medina Rodríguez JM, Fernández Alvarez CR, Sánchez García J, Fernández Carreira JM, Alvarez Martínez MV, Coto García E. [Lowering high levels of fasting total homocysteine with folic acid and vitamins B in patients with venous thromboembolism: relationship between response and the C677T methylenetetrahydrofolate reductase (MTHRF) genotype]. Med Clin (Barc). 2000 Jan 15;114(1):7-12. Spanish. doi: 10.1016/s0025-7753(00)71172-9.
185. Rao H, Wu H, Yu Z, Huang Q. *APOE* Genetic Polymorphism rs7412 T/T Genotype May Be a Risk Factor for Essential Hypertension among Hakka People in Southern China. Int J Hypertens. 2022 Sep 14;2022:8145896. doi: 10.1155/2022/8145896.
186. Varela ML, Adamczuk YP, Forastiero RR, Martinuzzo ME, Cerrato GS, Pombo G, Carreras LO. Major and potential prothrombotic genotypes in a cohort of patients with venous thromboembolism. Thromb Res. 2001 Dec 1;104(5):317-24. doi: 10.1016/s0049-3848(01)00384-x.
187. Félix TM, Leistner S, Giugliani R. Metabolic effects and the methylenetetrahydrofolate reductase (MTHFR) polymorphism associated with neural tube defects in southern Brazil. Birth Defects Res A Clin Mol Teratol. 2004 Jul;70(7):459-63. doi: 10.1002/bdra.20011.
188. Osunkalu VO, Taiwo IA, Makwe CC, Quao RA. Methylene tetrahydrofolate reductase and methionine synthase gene polymorphisms as genetic determinants of pre-eclampsia. Pregnancy Hypertens. 2020 Apr;20:7-13. doi: 10.1016/j.preghy.2020.02.001.
189. Messedi M, Frigui M, Chaabouni Kh, Turki M, Neifer M, Lahiyani A, Messaouad M, Bahloul Z, Ayedi F, Jamoussi K. Methylenetetrahydrofolate reductase C677T and A1298C polymorphisms and variations of homocysteine concentrations in patients with Behcet's disease. Gene. 2013 Sep 15;527(1):306-10. doi: 10.1016/j.gene.2013.06.041.
190. Yin G, Ming H, Zheng X, Xuan Y, Liang J, Jin X. Methylenetetrahydrofolate reductase C677T gene polymorphism and colorectal cancer risk: A case-control study. Oncol Lett. 2012 Aug;4(2):365-369. doi: 10.3892/ol.2012.740.
191. Fekih-Mrissa N, Mrad M, Ibrahim H, Akremi I, Sayeh A, Jaidane A, Ouertani H, Zidi B, Gritli N. Methylenetetrahydrofolate Reductase (MTHFR) (C677T and A1298C) Polymorphisms and Vascular Complications in Patients with Type 2 Diabetes. Can J Diabetes. 2017 Aug;41(4):366-371. doi: 10.1016/j.jcjd.2016.11.007.
192. Soltanpour MS, Soheili Z, Shakerizadeh A, Pourfathollah AA, Samiei S, Meshkani R, Shahjahani M, Karimi A. Methylenetetrahydrofolate reductase C677T mutation and risk of retinal vein thrombosis. J Res Med Sci. 2013 Jun;18(6):487-91.
193. Jiang S, Li J, Zhang Y, Venners SA, Tang G, Wang Y, Li Z, Xu X, Wang B, Huo Y. Methylenetetrahydrofolate reductase C677T polymorphism, hypertension and risk of stroke: a prospective, nested case-control study. Int J Neurosci. 2017 Mar;127(3):253-260. doi: 10.1080/00207454.2016.1183126.
194. Kumar M, Goudihalli S, Mukherjee K, Dhandapani S, Sandhir R. Methylenetetrahydrofolate reductase C677T variant and hyperhomocysteinemia in subarachnoid hemorrhage patients from India. Metab Brain Dis. 2018 Oct;33(5):1617-1624. doi: 10.1007/s11011-018-0268-5.
195. Karadeniz M, Erdogan M, Zengi A, Eroglu Z, Tamsel S, Olukman M, Saygili F, Yilmaz C. Methylenetetrahydrofolate reductase C677T gene polymorphism in Turkish patients with polycystic ovary syndrome. Endocrine. 2010 Aug;38(1):127-33. doi: 10.1007/s12020-010-9370-0.
196. Fang X, Namba H, Akamine S, Sugiyama K. Methylenetetrahydrofolate reductase gene polymorphisms in patients with cerebral hemorrhage. Neurol Res. 2005 Jan;27(1):73-6. doi: 10.1179/016164105X18313.
197. Safarinejad MR, Shafiei N, Safarinejad S. Methylenetetrahydrofolate reductase (MTHFR) gene C677T, A1298C and G1793A polymorphisms: association with risk for clear cell renal cell carcinoma and tumour behaviour in men. Clin Oncol (R Coll Radiol). 2012 May;24(4):269-81. doi: 10.1016/j.clon.2011.03.005.
198. Munshi R, Panchal F, Kulkarni V, Chaurasia A. Methylenetetrahydrofolate reductase polymorphism in healthy volunteers and its correlation with homocysteine levels in patients with thrombosis. Indian J Pharmacol. 2019 Jul-Aug;51(4):248-254. doi: 10.4103/ijp.IJP_215_19.
199. Chen L, Wu C, Dong Z, Cao S, Ren N, Yan X. Methylenetetrahydrofolate reductase polymorphisms and elevated plasma homocysteine levels in small vessel disease. Brain Behav. 2023 May;13(5):e2960. doi: 10.1002/brb3.2960.
200. Nakamura T, Saionji K, Hiejima Y, Hirayama H, Tago K, Takano H, Tajiri M, Hayashi K, Kawabata M, Funamizu M, Makita Y, Hata A. Methylenetetrahydrofolate reductase genotype, vitamin B12, and folate influence plasma homocysteine in hemodialysis patients. Am J Kidney Dis. 2002 May;39(5):1032-9. doi: 10.1053/ajkd.2002.32779.
201. Perez AB, D'Almeida V, Vergani N, de Oliveira AC, de Lima FT, Brunoni D. Methylenetetrahydrofolate reductase (MTHFR): incidence of mutations C677T and A1298C in Brazilian population and its correlation with plasma homocysteine levels in spina bifida. Am J Med Genet A. 2003 May 15;119A(1):20-5. doi: 10.1002/ajmg.a.10059.
202. Girelli D, Friso S, Trabetti E, Olivieri O, Russo C, Pessotto R, Faccini G, Pignatti PF, Mazzucco A, Corrocher R. Methylenetetrahydrofolate reductase C677T mutation, plasma homocysteine, and folate in subjects from northern Italy with or without angiographically documented severe coronary atherosclerotic disease: evidence for an important genetic-environmental interaction. Blood. 1998 Jun 1;91(11):4158-63.
203. Turaçli ME, Tekeli O, Ozdemir F, Akar N. Methylenetetrahydrofolate reductase 677 C-T and homocysteine levels in Turkish patients with pseudoexfoliation. Clin Exp Ophthalmol. 2005 Oct;33(5):505-8. doi: 10.1111/j.1442-9071.2005.01070.x.
204. Andreassi MG, Botto N, Cocci F, Battaglia D, Antonioli E, Masetti S, Manfredi S, Colombo MG, Biagini A, Clerico A. Methylenetetrahydrofolate reductase gene C677T polymorphism, homocysteine, vitamin B12, and DNA damage in coronary artery disease. Hum Genet. 2003 Feb;112(2):171-7. doi: 10.1007/s00439-002-0859-3.
205. Karabacak E, Aydin E, Ozcan O, Dogan B, Gultepe M, Cosar A, Muftuoglu T. Methylenetetrahydrofolate reductase (MTHFR) 677C>T gene polymorphism as a possible factor for reducing clinical severity of psoriasis. Int J Clin Exp Med. 2014 Mar 15;7(3):697-702.
206. Yuan RY, Sheu JJ, Yu JM, Hu CJ, Tseng IJ, Ho CS, Yeh CY, Hung YL, Chiang TR. Methylenetetrahydrofolate reductase polymorphisms and plasma homocysteine in levodopa-treated and non-treated Parkinson's disease patients. J Neurol Sci. 2009 Dec 15;287(1-2):64-8. doi: 10.1016/j.jns.2009.09.007.
207. Feng Z, Gao Y, Zhang M, Wang Y, Liu X, Zhang B, Su J, Wang H. Methylenetetrahydrofolate reductase (MTHFR) and methionine synthase reductase (MTRR) gene polymorphisms and five related serum molecular levels in 2587 patients: Associated differentially with adverse pregnancy. Mol Biol Rep. 2024 Sep 26;51(1):1014. doi: 10.1007/s11033-024-09948-x.
208. Kalita J, Srivastava R, Bansal V, Agarwal S, Misra UK. Methylenetetrahydrofolate reductase gene polymorphism in Indian stroke patients. Neurol India. 2006 Sep;54(3):260-3. doi: 10.4103/0028-3886.27148.
209. Siqueira ER, Oliveira CP, Muniz MT, Silva F, Pereira LM, Carrilho FJ. Methylenetetrahydrofolate reductase (MTHFR) C677T polymorphism and high plasma homocysteine in chronic hepatitis C (CHC) infected patients from the Northeast of Brazil. Nutr J. 2011 Aug 19;10:86. doi: 10.1186/1475-2891-10-86.
210. Huang T, Tucker KL, Lee YC, Crott JW, Parnell LD, Shen J, Smith CE, Ordovas JM, Li D, Lai CQ. Methylenetetrahydrofolate reductase variants associated with hypertension and cardiovascular disease interact with dietary polyunsaturated fatty acids to modulate plasma homocysteine in puerto rican adults. J Nutr. 2011 Apr 1;141(4):654-9. doi: 10.3945/jn.110.134353.
211. Fekih-Mrissa N, Mrad M, Klai S, Mansour M, Nsiri B, Gritli N, Mrissa R. Methylenetetrahydrofolate reductase (C677T and A1298C) polymorphisms, hyperhomocysteinemia, and ischemic stroke in Tunisian patients. J Stroke Cerebrovasc Dis. 2013 May;22(4):465-9. doi: 10.1016/j.jstrokecerebrovasdis.2013.03.011.
212. Franco Brochado MJ, Domenici FA, Candolo Martinelli Ade L, Zucoloto S, de Carvalho da Cunha SF, Vannucchi H. Methylenetetrahydrofolate reductase gene polymorphism and serum homocysteine levels in nonalcoholic fatty liver disease. Ann Nutr Metab. 2013;63(3):193-9. doi: 10.1159/000353139.
213. Arauz A, Hoyos L, Cantú C, Jara A, Martínez L, García I, Fernández Mde L, Alonso E. Mild hyperhomocysteinemia and low folate concentrations as risk factors for cervical arterial dissection. Cerebrovasc Dis. 2007;24(2-3):210-4. doi: 10.1159/000104479.
214. Mansoori N, Tripathi M, Luthra K, Alam R, Lakshmy R, Sharma S, Arulselvi S, Parveen S, Mukhopadhyay AK. MTHFR (677 and 1298) and IL-6-174 G/C genes in pathogenesis of Alzheimer's and vascular dementia and their epistatic interaction. Neurobiol Aging. 2012 May;33(5):1003.e1-8. doi: 10.1016/j.neurobiolaging.2011.09.018.
215. Silvestre GDS, Carrara IM, Flauzino T, Lozovoy MAB, Cecchini R, Reiche EMV, Simão ANC. *MTHFR* 677C>T (rsRS1801133) variant is associated with hyperhomocysteinemia but not with clinical severity in patients with peripheral arterial disease. J Vasc Bras. 2023 Nov 20;22:e20220061. doi: 10.1590/1677-5449.202200612.
216. Poduri A, Mukherjee D, Sud K, Kohli HS, Sakhuja V, Khullar M. MTHFR A1298C polymorphism is associated with cardiovascular risk in end stage renal disease in North Indians. Mol Cell Biochem. 2008 Jan;308(1-2):43-50. doi: 10.1007/s11010-007-9610-7.
217. Zidan HE, Rezk NA, Mohammed D. MTHFR C677T and A1298C gene polymorphisms and their relation to homocysteine level in Egyptian children with congenital heart diseases. Gene. 2013 Oct 15;529(1):119-24. doi: 10.1016/j.gene.2013.07.053.
218. Li MN, Wang HJ, Zhang NR, Xuan L, Shi XJ, Zhou T, Chen B, Zhang J, Li H. MTHFR C677T gene polymorphism and the severity of coronary lesions in acute coronary syndrome. Medicine (Baltimore). 2017 Dec;96(49):e9044. doi: 10.1097/MD.0000000000009044.
219. Li Z, Wu X, Huang H, Xu F, Liang G, Lin C, Qin Q, Lei X, Zeng X, Jiang X, Wei X. MTHFR C677T polymorphism and cerebrovascular lesions in elderly patients with CSVD: A correlation analysis. Front Genet. 2022 Sep 23;13:987519. doi: 10.3389/fgene.2022.987519.
220. Nursal AF, Kaya S, Sezer O, Karakus N, Yigit S. MTHFR gene C677T and A1298C variants are associated with FMF risk in a Turkish cohort. J Clin Lab Anal. 2018 Feb;32(2):e22259. doi: 10.1002/jcla.22259.
221. Zhang L, Fu H, Wei T. MTHFR gene polymorphism and homocysteine levels in spontaneous abortion of pregnant women. Am J Transl Res. 2021 Jun 15;13(6):7083-7088.
222. Biselli PM, Guerzoni AR, Goloni-Bertollo EM, Godoy MF, Abou-Chahla JA, Pavarino-Bertelli EC. [MTHFR genetic variability on coronary artery disease development]. Rev Assoc Med Bras (1992). 2009 May-Jun;55(3):274-8. Portuguese. doi: 10.1590/s0104-42302009000300018.
223. Sniezawska A, Dorszewska J, Rozycka A, Przedpelska-Ober E, Lianeri M, Jagodzinski PP, Kozubski W. MTHFR, MTR, and MTHFD1 gene polymorphisms compared to homocysteine and asymmetric dimethylarginine concentrations and their metabolites in epileptic patients treated with antiepileptic drugs. Seizure. 2011 Sep;20(7):533-40. doi: 10.1016/j.seizure.2011.04.001.
224. Guo J, Hao X, Wang R, Lian K, Jiang J, Chen N, Feng Z, Rao Y. *MTHFR* polymorphism's influence on the clinical features and therapeutic effects in patients with migraine: An observational study. Front Neurol. 2022 Dec 23;13:1074857. doi: 10.3389/fneur.2022.1074857.
225. Födinger M, Mannhalter C, Wölfl G, Pabinger I, Müller E, Schmid R, Hörl WH, Sunder-Plassmann G. Mutation (677 C to T) in the methylenetetrahydrofolate reductase gene aggravates hyperhomocysteinemia in hemodialysis patients. Kidney Int. 1997 Aug;52(2):517-23. doi: 10.1038/ki.1997.362.
226. Crott J, Thomas P, Fenech M. Normal human lymphocytes exhibit a wide range of methionine-dependency which is related to altered cell division but not micronucleus frequency. Mutagenesis. 2001 Jul;16(4):317-22. doi: 10.1093/mutage/16.4.317.
227. Candito M, Rivet R, Herbeth B, Boisson C, Rudigoz RC, Luton D, Journel H, Oury JF, Roux F, Saura R, Vernhet I, Gaucherand P, Muller F, Guidicelli B, Heckenroth H, Poulain P, Blayau M, Francannet C, Roszyk L, Brustié C, Staccini P, Gérard P, Fillion-Emery N, Guéant-Rodriguez RM, Van Obberghen E, Guéant JL. Nutritional and genetic determinants of vitamin B and homocysteine metabolisms in neural tube defects: a multicenter case-control study. Am J Med Genet A. 2008 May 1;146A(9):1128-33. doi: 10.1002/ajmg.a.32199.
228. Dorszewska J, Florczak J, Rozycka A, Kempisty B, Jaroszewska-Kolecka J, Chojnacka K, Trzeciak WH, Kozubski W. Oxidative DNA damage and level of thiols as related to polymorphisms of MTHFR, MTR, MTHFD1 in Alzheimer's and Parkinson's diseases. Acta Neurobiol Exp (Wars). 2007;67(2):113-29. doi: 10.55782/ane-2007-1639.
229. Ubeda N, Reyes L, González-Medina A, Alonso-Aperte E, Varela-Moreiras G. Physiologic changes in homocysteine metabolism in pregnancy: a longitudinal study in Spain. Nutrition. 2011 Sep;27(9):925-30. doi: 10.1016/j.nut.2010.10.017.
230. Mislanova C, Martsenyuk O, Huppertz B, Obolenskaya M. Placental markers of folate-related metabolism in preeclampsia. Reproduction. 2011 Sep;142(3):467-76. doi: 10.1530/REP-10-0484.
231. Xu H, Liu C, Wang Q. Plaque image characteristics, hyperhomocysteinemia, and gene polymorphism of homocysteine metabolism-related enzyme (MTHFR C677T) in acute coronary syndrome. Cell Biochem Biophys. 2013 Jun;66(2):403-7. doi: 10.1007/s12013-012-9483-6.
232. Rassoul F, Richter V, Janke C, Purschwitz K, Klötzer B, Geisel J, Herrmann W. Plasma homocysteine and lipoprotein profile in patients with peripheral arterial occlusive disease. Angiology. 2000 Mar;51(3):189-96. doi: 10.1177/000331970005100302.
233. Creus M, Deulofeu R, Peñarrubia J, Carmona F, Balasch J. Plasma homocysteine and vitamin B12 serum levels, red blood cell folate concentrations, C677T methylenetetrahydrofolate reductase gene mutation and risk of recurrent miscarriage: a case-control study in Spain. Clin Chem Lab Med. 2013 Mar 1;51(3):693-9. doi: 10.1515/cclm-2012-0452.
234. Yuan YG, Zhang ZJ, Li JJ. Plasma homocysteine but not MTHFR gene polymorphism is associated with geriatric depression in the Chinese population. Acta Neuropsychiatr. 2008 Oct;20(5):251-5. doi: 10.1111/j.1601-5215.2008.00290.x.
235. Minniti G, Calevo MG, Giannattasio A, Camicione P, Armani U, Lorini R, Piana G. Plasma homocysteine in patients with retinal vein occlusion. Eur J Ophthalmol. 2014 Sep-Oct;24(5):735-43. doi: 10.5301/ejo.5000426.
236. Bouaziz N, Ayedi I, Sidhom O, Kallel A, Rafrafi R, Jomaa R, Melki W, Feki M, Kaabechi N, El Hechmi Z. Plasma homocysteine in schizophrenia: determinants and clinical correlations in Tunisian patients free from antipsychotics. Psychiatry Res. 2010 Aug 30;179(1):24-9. doi: 10.1016/j.psychres.2010.04.008.
237. Nahar A, Sabo C, Chitlur M, Ravindranath Y, Lusher J, Rajpurkar M. Plasma homocysteine levels, methylene tetrahydrofolate reductase polymorphisms, and the risk of thromboembolism in children. J Pediatr Hematol Oncol. 2011 Jul;33(5):330-3. doi: 10.1097/MPH.0b013e318219324f.
238. Passaro A, Vanini A, Calzoni F, Alberti L, Zamboni PF, Fellin R, Solini A. Plasma homocysteine, methylenetetrahydrofolate reductase mutation and carotid damage in elderly healthy women. Atherosclerosis. 2001 Jul;157(1):175-80. doi: 10.1016/s0021-9150(00)00696-1.
239. de Carvalho SC, Muniz MT, Siqueira MD, Siqueira ER, Gomes AV, Silva KA, Bezerra LC, D'Almeida V, de Oliveira CP, Pereira LM. Plasmatic higher levels of homocysteine in non-alcoholic fatty liver disease (NAFLD). Nutr J. 2013 Apr 2;12:37. doi: 10.1186/1475-2891-12-37.
240. Palep-Singh M, Picton HM, Yates ZR, Barth J, Balen AH. Polycystic ovary syndrome and the single nucleotide polymorphisms of methylenetetrahydrofolate reductase: a pilot observational study. Hum Fertil (Camb). 2007 Mar;10(1):33-41. doi: 10.1080/14647270600950157.
241. Palep-Singh M, Picton HM, Yates ZR, Barth J, Balen AH. Polycystic ovary syndrome and the single nucleotide polymorphisms of methylenetetrahydrofolate reductase: a pilot observational study. Hum Fertil (Camb). 2007 Mar;10(1):33-41. doi: 10.1080/14647270600950157.
242. Sabino A, Fernandes AP, Lima LM, Ribeiro DD, Sousa MO, de Castro Santos ME, Mota AP, Dusse LM, das Graças Carvalho M. Polymorphism in the methylenetetrahydrofolate reductase (C677T) gene and homocysteine levels: a comparison in Brazilian patients with coronary arterial disease, ischemic stroke and peripheral arterial obstructive disease. J Thromb Thrombolysis. 2009 Jan;27(1):82-7. doi: 10.1007/s11239-007-0172-z.
243. Song J, Hou J, Zhao Q, Liu X, Guo Q, Yin D, Song Y, Li X, Wang S, Wang X, Duan J. Polymorphism of MTHFR C677T Gene and the Associations with the Severity of Essential Hypertension in Northern Chinese Population. Int J Hypertens. 2020 Oct 14;2020:1878917. doi: 10.1155/2020/1878917.
244. Sun JZ, Xu Y, Lu H, Zhu Y. Polymorphism of the methylenetetrahydrofolate reductase gene association with homocysteine and ischemic stroke in type 2 diabetes. Neurol India. 2009 Sep-Oct;57(5):589-93. doi: 10.4103/0028-3886.57808.
245. Lupi-Herrera E, Soto-López ME, Lugo-Dimas AJ, Núñez-Martínez ME, Gamboa R, Huesca-Gómez C, Sierra-Galán LM, Guarner-Lans V. Polymorphisms C677T and A1298C of MTHFR Gene: Homocysteine Levels and Prothrombotic Biomarkers in Coronary and Pulmonary Thromboembolic Disease. Clin Appl Thromb Hemost. 2019 Jan-Dec;25:1076029618780344. doi: 10.1177/1076029618780344.
246. Aléssio AC, Annichino-Bizzacchi JM, Bydlowski SP, Eberlin MN, Vellasco AP, Höehr NF. Polymorphisms in the methylenetetrahydrofolate reductase and methionine synthase reductase genes and homocysteine levels in Brazilian children. Am J Med Genet A. 2004 Jul 30;128A(3):256-60. doi: 10.1002/ajmg.a.30108.
247. Akoglu B, Kindl P, Weber N, Trojan J, Caspary WF, Faust D. Polymorphisms in the methylenetetrahydrofolate reductase gene are determinant for vascular complications after liver transplantation. Eur J Clin Nutr. 2008 Mar;62(3):430-5. doi: 10.1038/sj.ejcn.1602699.
248. Chedraui P, Salazar-Pousada D, Villao A, Escobar GS, Ramirez C, Hidalgo L, Pérez-López FR, Genazzani A, Simoncini T. Polymorphisms of the methylenetetrahydrofolate reductase gene (C677T and A1298C) in nulliparous women complicated with preeclampsia. Gynecol Endocrinol. 2014 May;30(5):392-6. doi: 10.3109/09513590.2014.895807.
249. Mantjoro EM, Toyota K, Kanouchi H, Kheradmand M, Niimura H, Kuwabara K, Nakahata N, Ogawa S, Shimatani K, Kairupan TS, Nindita Y, Ibusuki R, Nerome Y, Owaki T, Maenohara S, Takezaki T. Positive Association of Plasma Homocysteine Levels with Cardio-Ankle Vascular Index in a Prospective Study of Japanese Men from the General Population. J Atheroscler Thromb. 2016 Jun 1;23(6):681-91. doi: 10.5551/jat.32243.
250. Kim H, Park J, Chae H, Lee GD, Lee SY, Lee JM, Oh YS, Kim M, Kim Y. Potential Risk Factors Associated With Vascular Diseases in Patients Receiving Treatment for Hypertension. Ann Lab Med. 2016 May;36(3):215-22. doi: 10.3343/alm.2016.36.3.215.
251. Xu Y, Feng H, Zhang L, Li Y, Chi F, Ren L. Prevalence and clinical correlates of hyperhomocysteinemia in Chinese urban population with hypertension. Front Endocrinol (Lausanne). 2024 Feb 20;15:1369997. doi: 10.3389/fendo.2024.1369997.
252. Ibrahim S, El Dessokiy O. Prevalence of methylenetetrahydrofolate gene (MTHFR) C677T polymorphism among chronic hemodialysis patients and its association with cardiovascular disease: a cross-sectional analysis. Clin Exp Nephrol. 2009 Oct;13(5):501-507. doi: 10.1007/s10157-009-0194-2.
253. Ozarda Y, Sucu DK, Hizli B, Aslan D. Rate of T alleles and TT genotype at MTHFR 677C->T locus or C alleles and CC genotype at MTHFR 1298A->C locus among healthy subjects in Turkey: impact on homocysteine and folic acid status and reference intervals. Cell Biochem Funct. 2009 Dec;27(8):568-77. doi: 10.1002/cbf.1610.
254. Ulvik A, Hustad S, McCann A, Midttun Ø, Nygård OK, Ueland PM. Ratios of One-Carbon Metabolites Are Functional Markers of B-Vitamin Status in a Norwegian Coronary Angiography Screening Cohort. J Nutr. 2017 Jun;147(6):1167-1173. doi: 10.3945/jn.116.244657.
255. Zhang C, Xin QP, Xie YB, Guo XY, Xing EH, Dou ZJ, Zhao C. Relationship between methylenetetrahydrofolate reductase C677T gene polymorphism and neutrophil gelatinase-associated lipocalin in early renal injury in H-type hypertension. BMC Cardiovasc Disord. 2024 Jan 18;24(1):55. doi: 10.1186/s12872-024-03704-6.
256. Tang O, Wu J, Qin F. Relationship between methylenetetrahydrofolate reductase gene polymorphism and the coronary slow flow phenomenon. Coron Artery Dis. 2014 Dec;25(8):653-7. doi: 10.1097/MCA.0000000000000151.
257. Hu XJ, Su MR, Cao BW, Ou FB, Yin RX, Luo AD. Relationship between the methylenetetrahydrofolate reductase (MTHFR) rs1801133 SNP and serum homocysteine levels of Zhuang hypertensive patients in the central region of Guangxi. Clin Hypertens. 2023 Oct 1;29(1):26. doi: 10.1186/s40885-023-00250-9.
258. Zhang YD, Ke XY, Shen W, Liu Y. Relationship of homocysteine and gene polymorphisms of its related metabolic enzymes with Alzheimer's disease. Chin Med Sci J. 2005 Dec;20(4):247-51.
259. Sukla KK, Jaiswal SK, Rai AK, Mishra OP, Gupta V, Kumar A, Raman R. Role of folate-homocysteine pathway gene polymorphisms and nutritional cofactors in Down syndrome: A triad study. Hum Reprod. 2015 Aug;30(8):1982-93. doi: 10.1093/humrep/dev126.
260. Coppola G, Ingrosso D, Operto FF, Signoriello G, Lattanzio F, Barone E, Matera S, Verrotti A. Role of folic acid depletion on homocysteine serum level in children and adolescents with epilepsy and different MTHFR C677T genotypes. Seizure. 2012 Jun;21(5):340-3. doi: 10.1016/j.seizure.2012.02.011.
261. Gupta SK, Kotwal J, Kotwal A, Dhall A, Garg S. Role of homocysteine & MTHFR C677T gene polymorphism as risk factors for coronary artery disease in young Indians. Indian J Med Res. 2012 Apr;135(4):506-12. Erratum in: Indian J Med Res. 2013 Oct;138(4):574.
262. Safarinejad MR, Safarinejad S, Shafiei N. Role of methylenetetrahydrofolate reductase gene polymorphisms (C677T, A1298C, and G1793A) in the development of early onset vasculogenic erectile dysfunction. Arch Med Res. 2010 Aug;41(6):410-22. doi: 10.1016/j.arcmed.2010.08.005.
263. Wang Y, Wang Y, Sun Y, Zhang N, Liang X, Luo S, Dai L, Sun C, Yang Y, Li S, Zhang X, Zhang Q. Serum folate mediates the associations of *MTHFR* rs1801133 polymorphism with blood glucose levels and gestational diabetes mellitus in Chinese Han pregnant women. Br J Nutr. 2023 Oct 28;130(8):1329-1337.
264. Yasar A, Gunduz K, Onur E, Calkan M. Serum homocysteine, vitamin B12, folic acid levels and methylenetetrahydrofolate reductase (MTHFR) gene polymorphism in vitiligo. Dis Markers. 2012;33(2):85-9. doi: 10.3233/DMA-2012-0908.
265. Komlósi V, Hitre E, Pap E, Adleff V, Réti A, Székely E, Bíró A, Rudnai P, Schoket B, Müller J, Tóth B, Ottó S, Kásler M, Kralovánszky J, Budai B. SHMT1 1420 and MTHFR 677 variants are associated with rectal but not colon cancer. BMC Cancer. 2010 Oct 4;10:525. doi: 10.1186/1471-2407-10-525.
266. Yuan H, Fu M, Yang X, Huang K, Ren X. Single nucleotide polymorphism of *MTHFR* rs1801133 associated with elevated Hcy levels affects susceptibility to cerebral small vessel disease. PeerJ. 2020 Feb 20;8:e8627. doi: 10.7717/peerj.8627.
267. Ni J, Liu Y, Zhou T, Wu X, Wang X. Single Nucleotide Polymorphisms in Key One-Carbon Metabolism Genes and Their Association with Blood Folate and Homocysteine Levels in a Chinese Population in Yunnan. Genet Test Mol Biomarkers. 2018 Mar;22(3):193-198. doi: 10.1089/gtmb.2017.0195.
268. Carrizzo A, Iside C, Nebbioso A, Carafa V, Damato A, Sciarretta S, Frati G, Di Nonno F, Valenti V, Ciccarelli M, Venturini E, Scioli M, Di Pietro P, Bucci T, Giudice V, Storto M, Serio B, Puca AA, Giugliano G, Trimarco V, Izzo R, Trimarco B, Selleri C, Altucci L, Vecchione C. SIRT1 pharmacological activation rescues vascular dysfunction and prevents thrombosis in MTHFR deficiency. Cell Mol Life Sci. 2022 Jul 11;79(8):410. doi: 10.1007/s00018-022-04429-5.
269. Shi H, Yang S, Lin N, Huang P, Yu R, Chen M, Wang L, Jiang Z, Sun X. Study on Maternal SNPs of MTHFR Gene and HCY Level Related to Congenital Heart Diseases. Pediatr Cardiol. 2021 Jan;42(1):42-46. doi: 10.1007/s00246-020-02449-1.
270. Rashed L, Abdel Hay R, AlKaffas M, Ali S, Kadry D, Abdallah S. Studying the association between methylenetetrahydrofolate reductase (MTHFR) 677 gene polymorphism, cardiovascular risk and lichen planus. J Oral Pathol Med. 2017 Nov;46(10):1023-1029. doi: 10.1111/jop.12588.
271. Wilmanns C, Casey A, Schinzel H, Walter PK. Superficial thrombophlebitis in varicose vein disease: the particular role of methylenetetrahydrofolate reductase. Phlebology. 2011 Jun;26(4):135-9. doi: 10.1258/phleb.2009.009075.
272. Ma L, Jiang Y, Kong X, Yan M, Zhao T, Zhao H, Liu Q, Zhang H, Cao Y, Li P. Synergistic Effect of the *MTHFR* C677T and *EPHX2* G860A Polymorphism on the Increased Risk of Ischemic Stroke in Chinese Type 2 Diabetic Patients. J Diabetes Res. 2017;2017:6216205. doi: 10.1155/2017/6216205.
273. Xiang T, Xiang H, Yan M, Yu S, Horwedel MJ, Li Y, Zeng Q. Systemic risk factors correlated with hyperhomocysteinemia for specific *MTHFR C677T* genotypes and sex in the Chinese population. Ann Transl Med. 2020 Nov;8(21):1455. doi: 10.21037/atm-20-6587.
274. Ilhan N, Kucuksu M, Kaman D, Ilhan N, Ozbay Y. The 677 C/T MTHFR polymorphism is associated with essential hypertension, coronary artery disease, and higher homocysteine levels. Arch Med Res. 2008 Jan;39(1):125-30. doi: 10.1016/j.arcmed.2007.07.009.
275. Ferlazzo N, Gorgone G, Caccamo D, Currò M, Condello S, Pisani F, Vernieri F, Rossini PM, Ientile R. The 894G > T (Glu298Asp) variant in the endothelial NOS gene and MTHFR polymorphisms influence homocysteine levels in patients with cognitive decline. Neuromolecular Med. 2011 Sep;13(3):167-74. doi: 10.1007/s12017-011-8148-8.
276. Chen C, Gan YY. The allele frequencies of three polymorphisms in genes involved in homocysteine metabolism in a group of unrelated healthy Singaporeans. Dis Markers. 2010;29(2):111-9. doi: 10.3233/DMA-2010-0741.
277. Liang Z, Li K, Chen H, Jia J, Li J, Huo Y, Fan F, Zhang Y. The Association of Plasma Homocysteine Concentrations with a 10-Year Risk of All-Cause and Cardiovascular Mortality in a Community-Based Chinese Population. Nutrients. 2024 Jun 19;16(12):1945. doi: 10.3390/nu16121945.
278. Lee BH, Cheong HI, Shin YS, Cho BK, Wang KC. The effect of C677T mutation of methylene tetrahydrofolate reductase gene and plasma folate level on hyperhomocysteinemia in patients with meningomyelocele. Childs Nerv Syst. 2000 Sep;16(9):559-63. doi: 10.1007/PL00007298.
279. Liang S, Zhou Y, Wang H, Qian Y, Ma D, Tian W, Persaud-Sharma V, Yu C, Ren Y, Zhou S, Li X. The effect of multiple single nucleotide polymorphisms in the folic acid pathway genes on homocysteine metabolism. Biomed Res Int. 2014;2014:560183. doi: 10.1155/2014/560183.
280. Dinç N, Yücel SB, Taneli F, Sayın MV. The effect of the MTHFR C677T mutation on athletic performance and the homocysteine level of soccer players and sedentary individuals. J Hum Kinet. 2016 Jul 2;51:61-69. doi: 10.1515/hukin-2015-0171.
281. Rao J, Chen Y, Chen X, Wu R, Luo S, Lin Q, Dong Z, Huang J. The gene polymorphisms of eNOS and MTHFR modulates the development of preeclampsia in Han population. Heliyon. 2023 Nov 11;9(12):e22223. doi: 10.1016/j.heliyon.2023.e22223.
282. Chao CL, Tsai HH, Lee CM, Hsu SM, Kao JT, Chien KL, Sung FC, Lee YT. The graded effect of hyperhomocysteinemia on the severity and extent of coronary atherosclerosis. Atherosclerosis. 1999 Dec;147(2):379-86. doi: 10.1016/s0021-9150(99)00208-7.
283. Ho CH. The influence of age, sex, vitamin B(12), folate levels and methylenetetrahydrofolate reductase C677T genetic mutations on plasma homocysteine in the Chinese population. Haematologica. 2000 Oct;85(10):1051-4.
284. Cho SE, Hong KS, Shin GJ, Chung WS. The methylenetetrahydrofolate reductase C677T gene mutation is associated with hyperhomocysteinemia, cardiovascular disease and plasma B-type natriuretic peptide levels in Korea. Clin Chem Lab Med. 2006;44(9):1070-5. doi: 10.1515/CCLM.2006.194.
285. Nowak I, Bylińska A, Wilczyńska K, Wiśniewski A, Malinowski A, Wilczyński JR, Radwan P, Radwan M, Barcz E, Płoski R, Motak-Pochrzęst H, Banasik M, Sobczyński M, Kuśnierczyk P. The methylenetetrahydrofolate reductase c.c.677 C>T and c.c.1298 A>C polymorphisms in reproductive failures: Experience from an RSA and RIF study on a Polish population. PLoS One. 2017 Oct 26;12(10):e0186022. doi: 10.1371/journal.pone.0186022.
286. Stoccoro A, Tannorella P, Salluzzo MG, Ferri R, Romano C, Nacmias B, Siciliano G, Migliore L, Coppedè F. The Methylenetetrahydrofolate Reductase C677T Polymorphism and Risk for Late-Onset Alzheimer's disease: Further Evidence in an Italian Multicenter Study. J Alzheimers Dis. 2017;56(4):1451-1457. doi: 10.3233/JAD-161081.
287. Wang X, Zhou Y, Zhang M, Wang Y, Qin B. The methylenetetrahydrofolate reductase genotype 677CT and non-alcoholic fatty liver disease have a synergistic effect on the increasing homocysteine levels in subjects from Chongqing, China. Genes Dis. 2018 Jul 29;6(1):88-95. doi: 10.1016/j.gendis.2018.07.003.
288. Fukasawa M, Matsushita K, Kamiyama M, Mikami Y, Araki I, Yamagata Z, Takeda M. The methylentetrahydrofolate reductase C677T point mutation is a risk factor for vascular access thrombosis in hemodialysis patients. Am J Kidney Dis. 2003 Mar;41(3):637-42. doi: 10.1053/ajkd.2003.50125.
289. Carlus SJ, Abdallah AM, Bhaskar LV, Morsy MM, Al-Harbi GS, Al-Mazroea AH, Al-Harbi KM. The MTHFR C677T polymorphism is associated with mitral valve rheumatic heart disease. Eur Rev Med Pharmacol Sci. 2016;20(1):109-14.
290. Vallelunga A, Pegoraro V, Pilleri M, Biundo R, De Iuliis A, Marchetti M, Facchini S, Formento Dojot P, Antonini A. The MTHFR C677T polymorphism modifies age at onset in Parkinson's disease. Neurol Sci. 2014 Jan;35(1):73-7. doi: 10.1007/s10072-013-1545-z.
291. Hermans MP, Gala JL, Buysschaert M. The MTHFR CT polymorphism confers a high risk for stroke in both homozygous and heterozygous T allele carriers with Type 2 diabetes. Diabet Med. 2006 May;23(5):529-36. doi: 10.1111/j.1464-5491.2006.01841.x.
292. Qian XL, Cao H, Zhang J, Gu ZH, Tang WQ, Shen L, Hu JL, Yao ZF, Zhang L, Tang MN, Lv XC, Zhou J, Jin XJ, Hong B, Cui ZQ, Ge JB. The prevalence, relative risk factors and MTHFR C677T genotype of H type hypertension of the elderly hypertensives in Shanghai, China: a cross-section study : Prevalence of H type hypertension. BMC Cardiovasc Disord. 2021 Aug 4;21(1):376. doi: 10.1186/s12872-021-02151-x.
293. Murakami S, Matsubara N, Saitoh M, Miyakaw S, Shoji M, Kubo T. The relation between plasma homocysteine concentration and methylenetetrahydrofolate reductase gene polymorphism in pregnant women. J Obstet Gynaecol Res. 2001 Dec;27(6):349-52. doi: 10.1111/j.1447-0756.2001.tb01284.x.
294. Asanidze E, Tsetskhladze ZR, Kristesashvili J, Pirtskhalava M, Urjumelashvili M, Tchiokadze S, Vakhania M, Parunashvili N, Asanidze A. The Relationship between Homocysteine Levels, *MTHFR C677T and A1298C* Polymorphism, and Pregnancy Outcomes in Georgian Women with Polycystic Ovary Syndrome: A Case-Control Study. Int J Fertil Steril. 2025 Jan 5;19(1):50-57. doi: 10.22074/ijfs.2024.2008377.1504.
295. Wang F, Sui X, Xu N, Yang J, Zhao H, Fei X, Zhang Z, Luo Z, Xin Y, Qin B, Zhao X, Cao S, Zhang Y, Yang Z. The relationship between plasma homocysteine levels and MTHFR gene variation, age, and sex in Northeast China. Niger J Clin Pract. 2019 Mar;22(3):380-385. doi: 10.4103/njcp.njcp_291_18.
296. Wang F, Sui X, Xu N, Yang J, Zhao H, Fei X, Zhang Z, Luo Z, Xin Y, Qin B, Zhao X, Cao S, Zhang Y, Yang Z. The relationship between plasma homocysteine levels and MTHFR gene variation, age, and sex in Northeast China. Niger J Clin Pract. 2019 Mar;22(3):380-385. doi: 10.4103/njcp.njcp_291_18.
297. Mao X, Han L. The Relationship of Methylenetetrahydrofolate Reductase Gene C677T Polymorphism and Ischemic Stroke in Chinese Han Population. Ann Clin Lab Sci. 2018 Mar;48(2):242-247.
298. Loncar R, Müller BT, Zotz RB, Sucker C, Sandmann W, Scharf RE. The screening power of methylenetetrahydrofolate reductase C677T polymorphism versus plasma homocysteine concentration in patients with stenosis of the internal carotid artery. Thromb J. 2006 Sep 25;4:16. doi: 10.1186/1477-9560-4-16.
299. Marcucci R, Sofi F, Fedi S, Lari B, Sestini I, Cellai AP, Pulli R, Pratesi G, Pratesi C, Gensini GF, Abbate R. Thrombophilic risk factors in patients with severe carotid atherosclerosis. J Thromb Haemost. 2005 Mar;3(3):502-7. doi: 10.1111/j.1538-7836.2005.01173.x.
